# Common trust and personal safety issues: A systematic review on the acceptability of health and social interventions for persons with lived experience of homelessness

**DOI:** 10.1371/journal.pone.0226306

**Published:** 2019-12-30

**Authors:** Olivia Magwood, Vanessa Ymele Leki, Victoire Kpade, Ammar Saad, Qasem Alkhateeb, Akalewold Gebremeskel, Asia Rehman, Terry Hannigan, Nicole Pinto, Annie Huiru Sun, Claire Kendall, Nicole Kozloff, Emily J. Tweed, David Ponka, Kevin Pottie

**Affiliations:** 1 C.T. Lamont Primary Health Care Research Centre, Bruyère Research Institute, Ottawa, ON, Canada; 2 PET/CT Department, MyHealth Centre, Mississauga, ON, Canada; 3 Faculty of Medicine, McGill University Montreal, QC, Canada; 4 School of Epidemiology and Public Health, University of Ottawa, Ottawa, ON, Canada; 5 Department of Population Medicine, University of Guelph, Guelph, ON, Canada; 6 Department of Family Medicine & School of Epidemiology and Public Health, University of Ottawa, Ottawa, ON, Canada; 7 Ottawa Hospital Research Institute (OHRI), Ottawa, ON, Canada; 8 Li Ka Shing Knowledge Institute, St. Michael’s Hospital, Toronto, ON, Canada; 9 Centre for Addiction and Mental Health and Department of Psychiatry and Institute of Health Policy, Management and Evaluation, University of Toronto, Toronto, ON, Canada; 10 MRC/CSO Social and Public Health Sciences Unit, University of Glasgow, Glasgow, Scotland, United Kingdom; 11 Department of Family Medicine, University of Ottawa, Ottawa, ON, Canada; Università degli Studi di Perugia, ITALY

## Abstract

**Background:**

Persons experiencing homelessness and vulnerable housing or those with lived experience of homelessness have worse health outcomes than individuals who are stably housed. Structural violence can dramatically affect their acceptance of interventions. We carried out a systematic review to understand the factors that influence the acceptability of social and health interventions among persons with lived experience of homelessness.

**Methods:**

We searched through eight bibliographic databases and selected grey literature sources for articles that were published between 1994 and 2019. We selected primary studies that reported on the experiences of homeless populations interacting with practitioners and service providers working in permanent supportive housing, case management, interventions for substance use, income assistance, and women- and youth-specific interventions. Each study was independently assessed for its methodological quality. We used a framework analysis to identify key findings and used the GRADE-CERQual approach to assess confidence in the key findings.

**Findings:**

Our search identified 11,017 citations of which 35 primary studies met our inclusion criteria. Our synthesis highlighted that individuals were marginalized, dehumanized and excluded by their lived homelessness experience. As a result, trust and personal safety were highly valued within human interactions. Lived experience of homelessness influenced attitudes toward health and social service professionals and sometimes led to reluctance to accept interventions. Physical and structural violence intersected with low self-esteem, depression and homeless-related stigma. Positive self-identity facilitated links to long-term and integrated services, peer support, and patient-centred engagement.

**Conclusions:**

Individuals with lived experience of homelessness face considerable marginalization, dehumanization and structural violence. Practitioners and social service providers should consider anti-oppressive approaches and provide, refer to, or advocate for health and structural interventions using the principles of trauma-informed care. Accepting and respecting others as they are, without judgment, may help practitioners navigate barriers to inclusiveness, equitability, and effectiveness for primary care that targets this marginalized population.

## Introduction

Persons with lived experience of homelessness and vulnerable housing are an excluded population that is growing worldwide due to increasing costs of living, forces of urbanization,[1] and economic crises and their associated austerity policies.[2–4] Indeed, the number of individuals experiencing vulnerable housing conditions has reached 1.8 billion worldwide.[5] Over time, this population has struggled with mental health and addiction,[6] profound societal stigma, and structural violence, conditions which have led to high rates of treatment recidivism and inequitably low life expectancy rates.[7,8] Inadequate housing has been universally endorsed as a violation of human rights,[9] leading to international commitments to provide affordable and adequate housing services, within the next decade, through the 2030 Agenda for Sustainable Development.

“Structural violence” describes the social arrangements that put individuals and populations in harm's way through systematic exclusion over time.[10] These arrangements are considered structural because they are embedded in the political and economic organization of our social world; they are violent because they cause harm to people.[11] Structural violence manifests as unequal access to social systems of support like housing, health care, education and employment.[12] For example, the inability for individuals with “no fixed address” to receive coverage for healthcare or social services demonstrates how structural violence could stand in the way of addressing fundamental human needs.[13,14] Despite the impact of these structures on some of the most underserved patients, with few exceptions, primary care practitioners are not trained to influence such social forces[11] nor do they consistently have the capacity to adequately address their related social determinants of health [15]. To reduce structural violence and achieve health equity, practitioners and health systems must address the needs of underserved populations. Primary care providers have an opportunity to identify and act on social issues at the point of care by connecting patients with various support resources within and beyond the health system, such as local support groups, housing advocacy organizations or employment agencies.[16] Clinical practice guidelines represent an evidence-based tool designed to support providers’ decisions when delivering primary health care to persons with lived experience of homelessness. Such guidelines should consider the acceptability of the recommended community resources and interventions.

Interventions must emerge from local contexts and meet the essential needs and preferences of persons with lived experience of homelessness.[17–20] The content, scale and intensity of interventions should be proportionate to the level of social, economic or environmental disadvantage someone faces and the support they need.[21] Such interventions are complex in nature,[22–24] resulting in challenges to optimizing their design and delivery so as to guarantee the best health and social outcomes. There is wide agreement that a broad range of evidence is needed to inform decisions on the implementation of complex interventions, especially those that have impacts across the health or social care system.[25] Data from qualitative research contributes critical information to help decision-makers effectively address this need. The views of people experiencing social exclusion, such as homelessness, should be used to guide programs and practitioners so as to ensure that health and social services are inclusive, equitable, and effective.[17] As an example, many existing effective interventions have the potential to help the homeless and vulnerably housed forge routes out of homelessness.[17,26–28] However, the acceptability of these interventions is necessary for effectiveness, without this participants may fail to adhere to recommendations or benefit from improved outcomes.[29]

This review is one of a series of reviews to inform national clinical practice guidelines for the care of homeless or vulnerably housed persons. We conducted a Delphi consensus process that included 84 practitioners and 76 persons with lived experience of homelessness to prioritize relevant interventions which could be referred to by primary care providers, including permanent supportive housing, case management, income assistance, interventions for substance use, and women- and youth-focused interventions.[30] Our research team conducted parallel systematic reviews which evaluated the effectiveness of these interventions.[27,28,31–33] Given that health and structural interventions are affected by multiple intersecting factors that might not be captured in a quantitative review, the objective of this systematic review of qualitative studies is to understand the factors that influence the acceptability of these health and structural interventions from the perspective of persons who are homeless or vulnerably housed.

## Methods

### Search strategy and selection criteria

We conducted a systematic review according to a published protocol.[34] We included persons with lived experience of homelessness through all stages of this review so as to improve the relevance and applicability of the findings. Specifically, these individuals participated in the prioritization of selected interventions and populations of interest and in the interpretation of the qualitative key findings. We used the PRISMA guidelines to report our findings [35](See S1 File). Ethical approval was not required for this study.

Studies were eligible for inclusion in our systematic review if they utilized a qualitative design, involved data collection and analysis, and included at least one of the interventions specified in our protocol (S2 File). These reports could be published in any language. Mixed-method and quantitative studies were not eligible for inclusion as they are less likely to provide rich data compared with standalone qualitative studies.[36] We focused on studies whose participants were homeless or vulnerably housed, defined as individuals who are unsheltered, emergency sheltered, provisionally accommodated, or at risk of homelessness.[37] The subpopulations of interest included women, youth, and people with acquired brain injury or intellectual or physical disabilities, which were identified as priority groups in our Delphi consensus.[30] We excluded studies that focused exclusively on indigenous-specific interventions, as an examination of these interventions is being conducted by an indigenous-specific research team. The geographic setting was restricted to high-income countries [38] that have acknowledged affordable housing as a human right.[9] We excluded low-quality studies. (See S3 File for the full inclusion criteria).

We developed a systematic search for qualitative literature, using relevant keywords and MeSH terms (S4 File). We searched MEDLINE, EMBASE, PsycINFO, and ERIC via Ovid; and ProQuest Applied Social Sciences Index and Abstracts, Sociological Abstracts, Social Services Abstracts and Sociology Database for qualitative studies published from January 1^st^, 1994 to September 4^th^, 2019. We also searched grey literature for relevant studies and reports (S5 File). We did not hand-search for additional studies.

The identified studies were uploaded to Rayyan reference manager software to facilitate study selection.[39] Two reviewers independently screened titles/abstracts and full texts; disagreements were resolved through discussion or by consulting a third reviewer. We assessed the methodological quality of remaining studies by using the Critical Appraisal Skills Programme (CASP) checklist for qualitative studies.[40] CASP checklists are widely used in the health care domain and offer guidance for the critical appraisal with respect to trustworthiness, results and relevance of research studies. Criteria for the quality assessment of qualitative studies include the appraisal of a clearly stated aim, an appropriate qualitative methodology, and the consideration of ethical issues. We used the scoring system developed by Butler and colleagues (2016) to rate studies as high-, moderate-, or low-quality and report individual study assessments in the S6 File [41]. As done in other recent systematic reviews of qualitative studies [42–44] and in line with guidance available from the Cochrane Handbook[45], we excluded low-quality studies from the synthesis of our systematic review based on the outcome of our critical appraisal.

### Data analysis

We used a standardized data extraction sheet that included the study methodology, population, intervention, findings, study limitations, and funding details. Data were extracted by two reviewers independently. Disagreements were resolved through discussion.

We used the ‘best fit’ framework method as a systematic and flexible approach to analyzing the qualitative data.[46–48] Framework-based synthesis using the 'best fit' strategy is a highly pragmatic and useful approach for a range of policy urgent questions [49] whose use is supported in the development of clinical practice guidelines.[50] Framework synthesis is currently the most commonly used approach in a guideline process and is recommended when a question requires an understanding of complexity (see [50] for examples). Framework analysis is a five-stage process that includes familiarization with the data, identifying a thematic framework, indexing (applying the framework), charting and mapping, and interpretation.[51] Based on extensive team discussions, we selected the Risk and Vulnerability framework [52] due to its unique emphasis on structural factors, alongside biomedical and behavioural factors, that contribute to vulnerability and its stigma-informed approach. The Risk and Vulnerability framework identifies factors (causes of risk and vulnerability) and patient preferences that influence intervention accessibility, uptake, and acceptability. This framework additionally identifies biomedical, behavioural, and structural factors that act as enabling factors and influence corresponding causes of risk of and vulnerability to homelessness. A reviewer coded the data into the three domains of the Risk and Vulnerability framework, using a matrix spreadsheet to facilitate analysis; this was verified by a second reviewer (see Table 1). Mapping involved examining the concordant findings, disconformity data, and associations between themes. Interpretations were guided by our review objectives as well as the emerging themes. Mapping and interpretation were done through discussion with the entire review team.

**Table 1:** Risk and vulnerability framework levels [52].

FRAMEWORK LEVEL	DESCRIPTION
**Biomedical**	• Bodily states that can contribute to the development of chronic disease • May also be influenced by behavioural risk factors
**Behavioural**	• Individual lifestyle-related factors that influence health and may lead to the development or prevention of disease or disability • May include alcohol and drug use, inactivity, or care-seeking behaviours
**Structural**	• Environmental conditions that are outside of the control of individuals and that influence their perceptions, behaviours and health • Activities designed to alter specific environmental factors so as to create a more enabling environment for treatment, care and support • May include features of the social, cultural, economic, political and physical environment

We used the Confidence in the Evidence from Reviews of Qualitative research (CERQual) tool [25,53] to assess the confidence of the key findings of this review. We defined a key finding as the analytic output (e.g., theme or factor) from our qualitative evidence synthesis that, based on data from primary studies, described the experiences of persons engaging with health and structural interventions that target homeless populations.[53] CERQual bases the evaluation on four criteria: the methodological limitations of the included studies that support a review finding, the relevance of the included studies to the review question, the coherence of the review findings, and the adequacy of the data that contributes to a review finding (See Table 2).

**Table 2:** CERQual assessment components [25].

COMPONENT	Definition
**Methodological limitations**	The extent to which problems were identified in the way in which the primary studies which contributed to the evidence for a review finding were conducted
**Relevance**	The extent to which the primary studies supporting a review finding are applicable to the context specified in the review question
**Coherence**	The extent to which the pattern that constitutes a review finding is based on data that is similar across multiple individual studies and/or incorporates (compelling) explanations for any variations across individual studies
**Adequacy of data**	An overall determination of the degree of richness and/or scope of the evidence and quantity of data supporting a review finding

## Results

**Fig 1:**
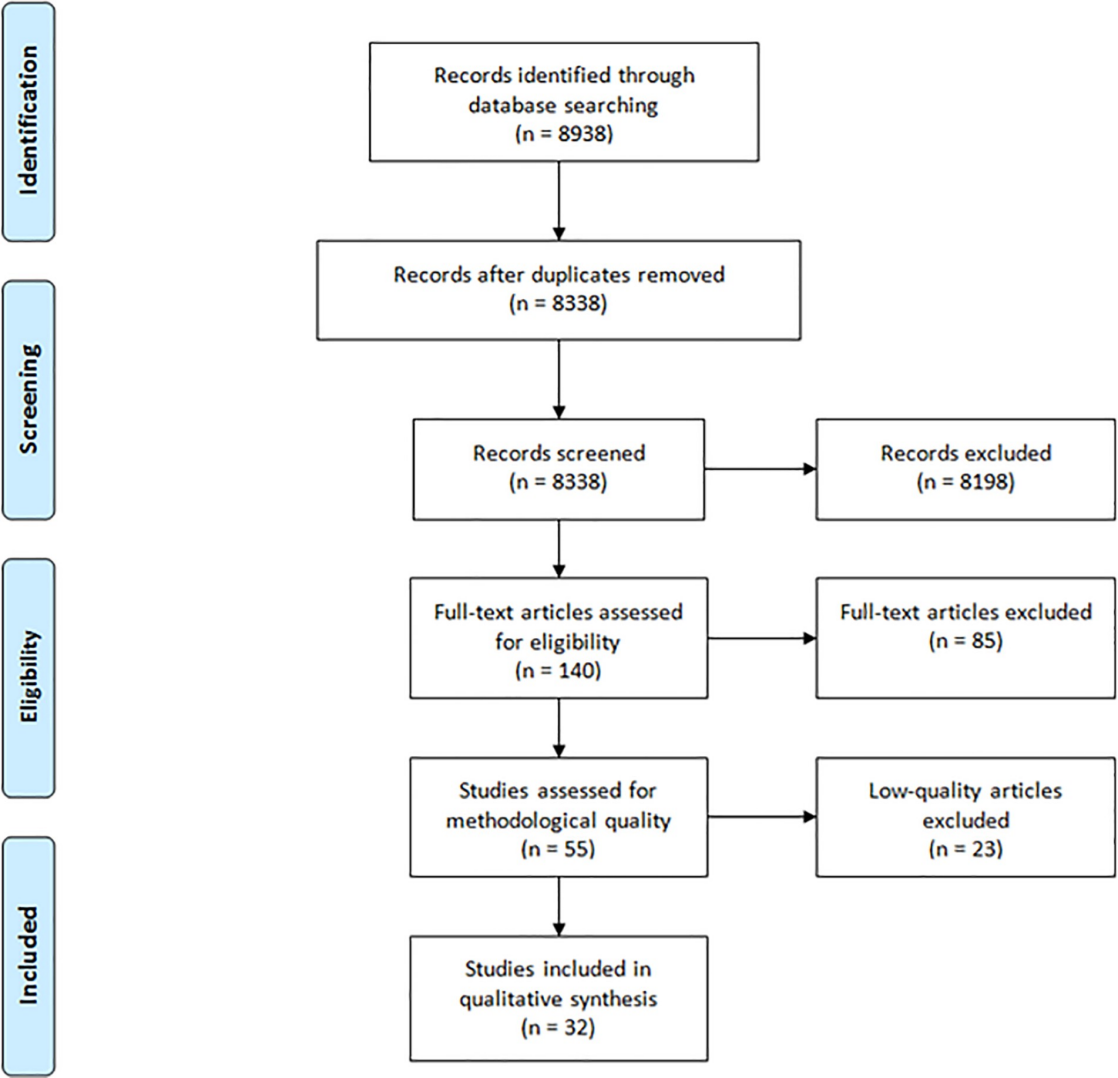
PRISMA flow diagram for original search up to 18^th^ January 2018.

**Fig 2:**
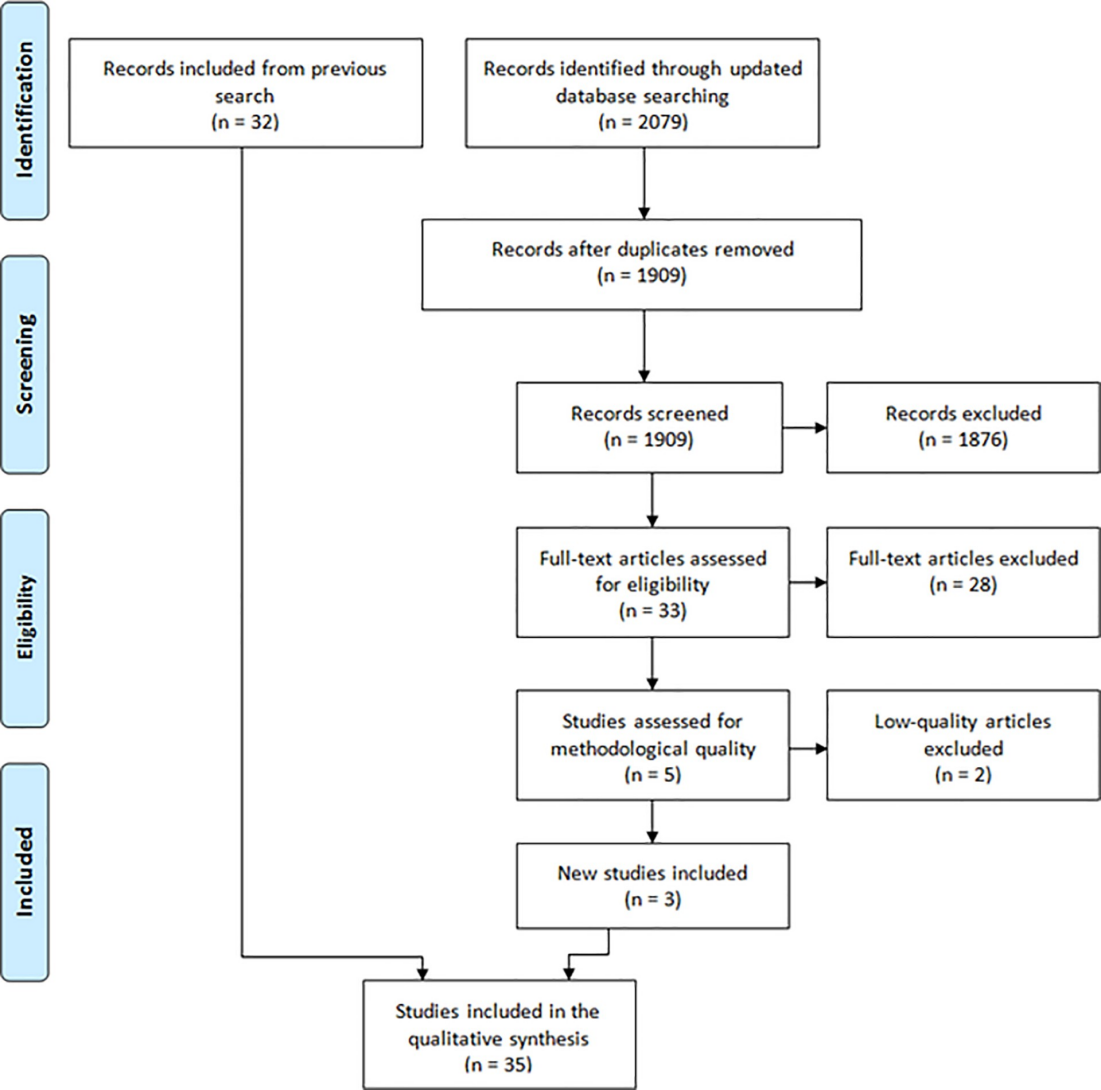
PRISMA flow diagram for updated search up to 4^th^ September 2019.

Our systematic search identified 11,017 citations. After deduplication, we screened 10,247 articles by title and abstract, excluded 10,074 articles and considered 173 studies for a full-text review, based on the relevance of the subject. The full-text review excluded a further 113 studies, leaving 60 studies for review, which were then assessed for methodological quality. Low-quality studies were excluded, leaving 35 studies that met our inclusion criteria and quality standards. (See Figs 1 and 2: PRISMA and S7 File).

Table 3 shows the characteristics of the included studies that are heterogeneous in terms of sample size, intervention, setting, and target populations. These studies represent the views of over 900 service users. Of the 35 included studies, 14 were conducted in Canada, 14 in the USA, 6 in the UK and 1 in Australia. Interventions included women-specific education and peer support, case management, place-based interventions for youth, non-abstinence-contingent supportive housing programs (e.g., Housing First), income assistance, needle-exchange services, and peer administration of naloxone. Target populations included youth, women, the elderly, men, mothers and families, and generalized populations who experienced homelessness and vulnerable housing. Table 3 presents the CASP summary of the methodological assessment (see also Appendix S6).

**Table 3:** Characteristics of included studies.

Study ID	Objective	Methodology	Population	Study setting and country	Sample size	Intervention	Quality assessment (CASP)
Alhusen et al. 2017 [54]	To explore the perceived benefits of participating in a mindfulness program in mother–child dyads.	In-depth qualitative interviews guided by open-ended questions with relevant probes. Interviews were analyzed by identification of categories and themes.	Homeless and vulnerably housed mothers receiving services at a therapeutic nursery serving homeless children under the age of 3 years	Therapeutic Nursery United States	n = 17 A convenience sample of predominantly African American mothers	The SHINE (Support, Honor, Inspire, Nurture, Evolve) mindfulness program is an innovative 10-session program that teaches mindfulness awareness practices to people living with homelessness. It includes an active-play component to strengthen mother-child attachment relationship, promote mutual regulation, and address trauma-induced developmental delays.	9/10
Archard & Murphy 2015 [55]	To explore the support work programme as it was received by service users domiciled in supported housing for homeless persons.	Narrative interviews with results analyzed thematically.	Homeless men residing in supported accommodation carrying a formal or suspected diagnosis of a trauma-related psychiatric condition and/or referral by statutory health services	Supported accommodation centre UK	n = 6 participants (4 service users and 2 support workers) A convenience sample of male service users of white British descent.	A support work programme introduced at a traumatic stress service to augment psychotherapeutic interventions. The service is provided by support workers involved in providing practical assistance with a range of everyday tasks, benefit and grant applications, seeking of accommodation with service users, home visits and meetings with family, liaison with other agencies, making referrals.	7.5/10
Aviles & Helfrich 2004 [56]	To learn the perspectives of homeless youth by exploring their needs, services they find helpful, and barriers to accessing services.	Qualitative semi-structured oral life history interviews. Transcripts were coded and then analyzed using constant comparative methods.	Homeless youth aged 14–21 admitted to an emergency-housing program.	New Opportunities Shelter United States	n = 30 A convenience sample of predominantly African American and Latino youth	Services at an emergency shelter for youth providing a case worker to assist residents in next steps in housing and self-sufficiency by addressing issues of schooling, job training, job searching, medical care, prenatal care, mental health services, substance abuse services, and family reunification.	7.5/10
Barker et al. 2018 [57]	To understand what participants believe are the critical factors for effective intentional peer support, through exploring participants' experiences of providing/receiving this support.	Semi-structured interviews with thematic analysis.	Homeless adults aged 18 and above who have provided or received intentional peer support.	Four organizations: (1)Organization providing peer support (2)Peer-led outreach service (3)Emergency accommodation (4)Emergency night shelter UK	n = 29 Predominantly male participants with experience of substance misuse identified through snowball sampling.	Peer mentors help clients to navigate complex health and social systems to access services, including benefits, housing, and job searches, and providing emotional support.	9/10
Canham et al, 2019 [58]	To understand the experience of Metro Vancouver’s Homelessness Partnering Strategy-funded Housing First (HF) program and how it is functioning from the perspective of a representative sample of providers and clients who deliver and receive HF services.	One-on-one interviews and focus groups. Using the SWOT (Strengths, Weaknesses, Opportunities, Threats) framework, the interview and focus group data were categorized and thematically analyzed.	Participants in a Housing First program aged 19 years and older.	Greater Vancouver Shelter Strategy (GVSS), a non-profit organization of shelter and outreach providers Canada	One-on-one interviews n = 26 Focus groups n = 8 Provider participants recruited by email, with a convenience sample of program clients.	Housing First (HF): Permanent supportive housing in both congregate and scattered-site models, includes support in the form of intensive case management or assertive community treatment.	9/10
Chaturvedi 2016 [59]	To understand young, homeless people’s personal experiences of counselling as well as their views on barriers and facilitators to accessing counselling.	Individual, face-to-face semi-structured interviews. Transcripts analyzed by thematic analysis.	Homeless young people aged 16–25 years	Homelessness charity UK	n = 6 A purposive sample of participants (four female, two male)	Charity serving homeless populations which provides supportive housing and provision of counselling to young people.	10/10
Collins et al. 2012 [60]	To explore the strengths and challenges of a project-based Housing First (HF) program and to elucidate potential points for program enhancement. To examine residents’ transitions into housing and the day-to-day experiences of residents and staff who live and work in a project-based HF program.	Naturalistic observation and document analysis followed by one-on-one semi-structured resident interviews and a single two-hour, semi-structured focus group with program staff. Field notes and transcripts were thematically analyzed.	Chronically homeless individuals with alcohol problems that are residents of a Housing First program	Project-based Housing First program United States	n = 17 Predominantly Caucasian and American Indian/Alaskan Native residents with experience of alcohol misuse.	Project-based Housing First (permanent supportive housing) for chronically homeless individuals with alcohol problems.	8/10
Cormack 2009 [61]	To explore young homeless people’s views of counselling, with a view to considering how the counselling profession can better meet their needs.	Mini-group focus groups were conducted and then analyzed with grounded theory methodology.	Homeless youth aged 16 to 21	Two residential projects for young homeless people UK	n = 8 The majority (four) were female, one of the young people was black, one Asian, and the rest white.	Counselling services offered within residential projects for homeless young people.	9/10
Davis et al. 2012 [62]	To understand what value the enrollees’ found in ongoing engagement with the intensive case management program; and which of the programs many interventions were perceived by their participant enrollees as effective in improving health.	In-depth one-on-one interviews using open-ended questions. Results were analyzed using the constant comparative methodology.	Homeless and vulnerably housed chronically ill persons considered “frequently admitted patients” (3 admissions in the previous 12 months)	A public urban teaching hospital l affiliated with the University of California, San Francisco United States	n = 14 Program enrollees (eight men and six women between 26 to 64 years; eight were African American, four were white, one was Latino, and one was Asian)	A publicly funded outpatient intensive case management program at a public urban teaching hospital. Each case manager conducted home visits with each of her 10–15 clients weekly or more frequently. The program staff assisted enrollees with medical issues, such as medication refills and appointments; social issues, such as obtaining housing and entitlements; and with drug and alcohol treatment.	10/10
Farquhar et al. 2014 [63]	To understand how consumers describe “success” and “recovery” in their own words and using their own examples, and to learn about the strengths and challenges of programs and services from the consumers’ perspectives	Individual in-depth interviews with thematic analysis	Vulnerably housed individuals that had participated in at least two programs or services including alcohol and drug free community housing, family housing, transitional housing, and Housing First housing units.	Central City Concern (CCC), an internationally recognized organization that provides housing, employment, recovery and health-related services for persons experiencing homelessness or at risk of homelessness. United States	n = 16 Participants who represented over a dozen different housing, health, recovery, and employment programs.	Alcohol and drug free community housing, family housing, transitional housing, and Housing First housing units	9.5/10
Ferguson & Islam. 2008 [64]	To explore clients’ perceptions of key changes in their lives as a result of participation in the Social Enterprise Intervention (SEI).	Summative focus-group interviews analyzed using the constant comparative method and identification of themes.	Homeless young adults aged 18–24 years	Drop-in centre for homeless youth United States	n = 5 Purposeful sampling was used to select information-rich participants (four male, one female).	Social Enterprise Intervention: Vocational intervention specifically designed for street-living young adults with mental health issues and limited service engagement.	10/10
Guilcher et al. 2016 [65]	To explore the experiences with health and social services of men who had histories of problem gambling and/or substance use problems and housing instability in an urban center.	Community-based participatory approach using qualitative semi-structured interviews and descriptive questionnaire with thematic analysis.	Men with gambling problems and/or substance use problems and housing instability	Good Shepherd Ministries, a community-based organization that provides services for men with housing instability. Canada	n = 30 Random sample of eligible participants identified by the local organization	Housing services, social assistance, programs for substance use and problem gambling.	9/10
Gultekin et al. 2014 [66]	To explore individual pathways into homelessness, understand the day-to-day experience of living in an emergency shelter and the process of rehousing, identify real and perceived barriers for families attempting to reestablish stable housing, and understand the impact of homelessness on families’ overall health and well-being.	Focus groups guided by semi-structured interviews, and content analysis, with qualitative thematic analysis.	Homeless mothers with at least one dependent child and their caseworkers.	An income assistance service agency. United States	n = 13 homeless mothers n = 5 caseworkers Caseworkers were asked directly to participate in the focus groups by the study project manager. Mothers were selected purposively	A service agency in that provides emergency financial assistance, job training, fiscal planning, life skills classes, and a wide array of social services to homeless families. One of the agency’s major programs is directed toward providing housing and employability services for homeless adults (primarily African American women), which include career development training and household management	9.5/10
Holtschneider 2016 [67]	To investigate the perceived utility transitional living programs as a housing model for youth experiencing homelessness.	In-depth semi-structured, open-ended interviews with thematic analysis and brief questionnaire.	Homeless youth aged 20 to 32 who exited a transitional living program.	Non-profit agency offering a transitional living program for homeless youth. United states	n = 32 A purposive sample of participants (19 female, 13 males, majority African American)	A transitional living program for homeless youth. Services provided include housing, counseling, life skills development, interpersonal skill building, educational advancement, job attainment skills, and mental and physical health care.	8.5/10
Jost et al. 2011 [68]	To provide a better understanding of clients’ perceptions of Street to Home (S2H; a housing-focused outreach program) operations, including its strengths and weaknesses	Semi-structured interviews analyzed with grounded theory and comparative analysis.	Homeless and vulnerably housed individuals who had been placed into permanent housing	Centre for Urban Community Services United States	n = 20 Participants who had been placed into permanent housing by S2H during the previous six months (18 men, 2 women)	Street to Home (S2H), a street outreach program whose primary aim is to place long term unsheltered homeless individuals directly into housing. Based on the principles of ‘housing first’, the program provides immediate access to transitional or permanent housing, bypassing time spent in shelters or drop-in centers.	10/10
Kozloff et al. 2013 [69]	To increase understanding of facilitators and barriers to service use among homeless youths with co-occurring disorders.	Focus groups guided by semi-structured interviews with thematic guided analysis.	Homeless youth aged 18–26 with co-occurring mental and substance use disorders.	Inner city agencies offering mental health services to homeless youth Canada	n = 23 Youths were recruited consecutively and were predominantly white males	Inner-city agencies offering variety of services for homeless youth. Agencies were selected to reflect a breadth of service users: a shelter with short- and long-term facilities where substances were banned on site, an emergency shelter that used a harm-reduction approach, and a drop-in center and health clinic that served many street-living youths who avoid shelters. At each agency, mental health and addictions services were available on site, including consulting psychiatrists.	9.5/10
Lamanna et al. 2018 [70]	To examine the role of a brief interdisciplinary intervention in supporting continuity of care.	Qualitative interviews and focus groups, and analyzed data using thematic analysis	Homeless adults discharged from hospital.	A partnership of three local hospitals, a large community mental health agency, a homeless shelter, a physician practice plan and a peer-run service organization. Canada	n = 52 Participants included programme service users (N = 22), programme staff (N = 8), managers of partnered organizations (N = 7), people with lived experience of homelessness (N = 8), and external service providers (N = 7).	Co-ordinated Access to Care for Homeless People (CATCH) provides brief (up to 6 months) case management and access to interdisciplinary care for homeless adults discharged from hospital and lacking appropriate health and social supports. Case managers assess needs, introduce service users to providers within the interdisciplinary team, offer assertive outreach and home visits, and facilitate connections to income supports, housing, and community-based health and social services as needed	9.5/10
Lorelle & Grothaus. 2015 [71]	To explore the experiences of children receiving services from a supportive housing programs as well as parents’ perceptions of how their children’s psychosocial needs are being met while receiving services from supportive housing programs.	Semi-structured interviews with inductive data analysis that allowed for themes to emerge naturally.	Homeless or at risk families and their children.	Urban area agency providing services to homeless families United States	n = 22 Participants (parents n = 9, children n = 13) were purposively sampled. All participants were female.	Emergency shelter, permanent or transitional supportive housing programs, and in-home case management program.	9.5/10
Macnaughton et al. 2016 [72]	To explore the role of housing first in promoting the recovery of people with mental illness who are homeless.	Narrative interviews using a modified version of constructivist grounded theory analysis.	Homeless individuals with mental illness	The At Home/ Chez Soi trial in five Canadian cities Canada	n = 195 119 participants received housing first, 76 received treatment-as-usual	The At Home/ Chez Soi project randomized participants into housing first (permanent supportive housing with intensive case management or assertive community treatment) or treatment as usual.	7.5/10
MacNeil & Pauly 2011 [73]	To explore the meaning of needle exchange programs from the perspective of people who use needle exchange services.	Observations, semi-structured interviews and focus groups. Qualitative description analysis was used to analyze the data.	Users of a needle exchange program, the majority of which were vulnerably housed	Four needle exchange sites Canada	n = 33 A convenience sample of participants (23 men, 10 women)	Needle Exchange Program	8/10
Magee et al. 2008 [74]	To examine the benefits and challenges of Ladies’ Night to inform direct service delivery and advocacy efforts for homeless and marginally housed women in San Francisco.	In-depth interviews, qualitative self-administered surveys and field notes. Data were analyzed according to themes.	Homeless and marginally housed women who have attended the intervention for at least 3 months.	The Mission Neighbourhood Resource Centre (MNRC) United States	(1) Interviews with 5 providers and 8 participants; (2) a self-administered survey completed by 7 participants; and (3) observation field notes from 9 Ladies’ Night sessions.	Ladies’ Night is a resource-rich drop-in program for homeless and marginally housed women that provides access to clean needles, direct referral to 60-day methadone detoxification, dinner, and safe sex and hygiene supplies in a community setting. Other services include chair massage, art activities, games, and a monthly clothing closet. Health education discussions are periodically held on topics ranging from safer sex to overdose prevention to reproductive health care.	8.5/10
McMaster et al. 2017 [75]	To explore the experiences of the clients, case managers and health professionals of the maintenance support program.	Qualitative descriptive methods incorporating face-to-face interviews that were thematically analyzed	Women at risk of homelessness who had participated in the maintenance support program for three months	Day-time drop-in maintenance support and respite centre Australia	n-21 n = 15 female clients n = 6 case managers and healthcare professionals	A maintenance support program (MSP) which provides support to women who are present in crisis. Many of the clients are homeless and are working through the effects of trauma and abuse together with mental and/or physical illnesses. The program offers case management and healthcare service provision together with activities for life skills within a supportive and safe environment.	7.5/10
Mitchell et al. 2017 [76]	To provide insights about how young adults perceive and interpret the effect that take home naloxone (THN) programs have upon them and their behaviors, and to explore how providing THN programming may influence their relationship with their health-care team.	Community-based participatory research through focus groups and individual interviews. Thematic analysis through consensus building.	Youth aged 19 to 25 who are homeless or precariously housed	Providence Health Care’s Inner City Youth (ICY) Program and Granville Youth Health Centre, Vancouver, Canada	n = 11 A purposive sample of young adults	Take home naloxone program which involved providing naloxone and teaching people how to prevent, recognize, and respond to an opioid overdose, including seeking professional help, rescue breathing and how to correctly administer naloxone, as a part of an intensive case management program for youth.	9.5/10
Patterson et al. 2013 [77]	To identify trajectories of recovery among homeless adults with mental illness alongside the factors that contribute to positive, negative, mixed or neutral trajectories over time.	Narrative interviews conducted at baseline and 18 months later analyzed by thematic analysis	Homeless adults with mental illness	Vancouver community residences (congregate housing or independent apartments) Canada	n = 43 Participants were randomly and purposively selected	Housing First with assertive community treatment or intensive case management	8/10
Patterson et al. 2014 [78]	To explore the changes (if any) participants perceived over 18 months post randomization [in an RCT of Housing First] and to understand factors that facilitated or hindered this change.	Semi-structured interviews with thematic analysis	Homeless adults with mental disorders	Vancouver Housing First Program Canada	n = 43 Participants were randomly and purposively selected	Housing First with intensive supports (assertive community treatment or intensive case management)	9/10
Perreault et al. 2016 [79]	To examine the perspectives of Prometheus residents based on their sources of satisfaction and dissatisfaction regarding the program, and to compare them with those emerging from the literature review on resident’s perspectives in other forms of transitional housing.	Focus groups and individual semi-structured interviews. Transcribed responses were divided into ‘‘concept units”, groupings of words or sentences corresponding to one concept, and then classified using a content analysis.	Vulnerably housed individuals receiving medical treatment for their opioid dependence.	Habitations Prometheus, a peer-run transitional housing program for opioid users Canada	n = 13 Focus group n = 7 Interviews n = 6	A day centre aiming to improve the health and well-being of opioid-dependent persons, including peer service providers who were selected to run the program and were required to have experience in providing help to others who struggle with similar problems.	8/10
Ploeg et al. 2008 [80]	To describe how the Homeless Intervention Program (HIP) addressed the needs of elderly people who were homeless or at risk of homelessness and to describe the factors that influenced the program.	Intrinsic case study approach using in-depth interviews, focus group interviews and client records and analyzed using a circular approach.	Elderly people who were homeless or at risk of homelessness.	Neighbourhood-based multiservice non-profit agency Canada	n = 28 Participants were purposively sampled across four participant groups: (1) HIP clients; (2) HIP service providers and the HIP administrator; (3) other service providers and administrators who collaborated with the HIP staff; and (4) local administrators of the homelessness funding programme.	The Homeless Intervention Program (HIP) providers used a case management approach to provide individually tailored services to vulnerable elderly people. This approach involved: (1) meeting with clients to determine their issues and needs; (2) negotiating a plan of care; (3) offering practical assistance; (4) making referrals to appropriate agencies and supports; (5) advocating for housing, health and income supports; (6) working collaboratively with other providers and agencies; and (7) providing follow-up over time.	8.5/10
Poremski et al. 2015 [81]	To investigate the way people with mental illness who are recently homeless experience supported employment services and how their experiences compare with those of participants receiving usual services.	Semi structured qualitative interviews. Responses analyzed thematically	Homeless and precariously housed individuals with presence of mental illness.	The At Home/ Chez Soi project and Individual Placement and Support (IPS) trial within scattered site housing Canada	n = 27 n = 14 received supported employment, n = 13 received usual services.	Housing First scattered-site housing first services, including services from intensive case management (ICM) teams and supported employment (IPS) services	7.5/10
Quinn et al. 2015 [82]	To examine the role of permanent supportive housing in the lives of HIV-positive mothers and their children to improve the case management and supportive services available in supportive housing programs.	In-depth semi-structured interviews analyzed for emergent themes using the principles of grounded theory analysis.	Female caregivers who were HIV positive living in permanent supportive housing	A partnership with a nonprofit agency providing safe and affordable housing for low-income individuals United States	n = 14 Participants included 10 female heads of household who were HIV-positive and residing in supportive housing units with their families. Additionally, 4 supportive housing program staff members were interviewed.	Supportive housing for homeless people living with HIV/AIDS following a Housing First and harm reduction model, wherein eligibility is not contingent on sobriety or a clean criminal record. Program included provision of case management.	9/10
Radey & Wilkins. 2010 [83]	To explore how service recipients and providers view employment prospects for homeless individuals and how community partnerships and services can help this vulnerable population secure jobs.	Semi structured open-ended interviews analyzed thematically using the principles of grounded theory.	Unemployed homeless adults who did not qualify for government benefits and were residing in a local shelter	Community partnership within a shelter United States	n = 35 n = 13 convenience sample of participants (10 men, 3 women) n = 22 people involved in the community partnership.	A community partnership that funded an employment specialist to provide an employment workshop and employment-focused, on-site case management for residents at local shelters.	7.5/10
Stewart et al. 2010 [84]	To explore support needs, support resources and coping strategies (e.g. support seeking) used by homeless youth as well as the strengths and weaknesses/gaps of support programs available to homeless youth and their preferences for support interventions.	Individual and group interviews analyzed using a coding framework which allowed for identification of themes and sub-themes.	Homeless youth between 15 and 25 years of age	Local agency providing services for homeless youth Canada	n = 62 Young adults (n = 35) and their service providers (n = 27)	Youth support programs and services accessible through agencies.	9/10
Taylor et al. 2007 [85]	To investigate young homeless people's experiences of "Strong Minded", a new mental health service set up within selected homeless shelters and run by a voluntary sector organization.	Semi-structured interviews with in-depth thematic analysis	Vulnerably housed residents of homeless shelters aged 16–23 years old who displayed mental health related problems	Five "Foyers" homeless shelters from the 18 which received the Strong Minded service. UK	n = 19 13 female and 6 males	"Strong Minded"—mental health intervention offered within shelters. This provided assessment, in-house short-term interventions, referral to other services, liaison with agencies and training.	9.5/10
Thompson et al. 2006 [86]	To understand the perceptions of homeless young people concerning their experienced with services and providers (characteristics of these services and providers that are helpful or unhelpful).	Semi-structured focus groups, audiotaped, transcribed verbatim and iteratively analyzed thematically.	Homeless youth and young adults, age 16–23 years, receiving health and social services from a community drop-in center	Community drop-in centre United States	n = 60 Convenience sample of homeless youth in 7 focus groups	Case management and mental health services, employment and financial assistance, place-based interventions for youth	9.5/10
Wright et al. 2006 [87]	To explore the relationship between acceptability to drug users, the degree of responsibility that drug users are prepared to take in the overdose situation, a description of possible risk entailed in introducing such a project and the inter-relationships between risk perception and possible benefits or harms of take home nalloxone amongst homeless drug users.	In depth face-to-face interviews. Interviews recorded, transcribed and analyzed thematically by framework techniques.	Individuals with past or current history of heroin use, past or current history of homelessness, and either personal experience or experience of a peer experiencing a heroin overdose.	One primary care centre and two non-statutory organizations UK	n = 27 A purposive sample of white Caucasian individuals (19 men and 8 women)	Peer-administration of naloxone	8.5/10
Yamin et al. 2014 [26]	To describe the program theory of peer supportive housing (PSH), as understood by tenants and program staff, to identify the challenges associated with the implementation of the PSH, to describe the strengths and weaknesses of the PSH as perceived by program staff and tenants, to examine the perceived impacts of the PSH, to provide suggestions for improvement of the PSH.	In-person interviews which were audio-taped and transcribed before thematic coding.	Current and former Peer Supportive Housing (PSH) tenants for individuals experiencing homeless and substance abuse/mental health issues.	A permanent supportive housing program with six large apartments Canada	n = 14 5 programs staff and 9 tenants	Housing First (HF) pilot program for HF participants who have experienced difficulty achieving housing stability, called Peer Supportive Housing (PSH). Peer support is offered to tenants by the peer support couple, in additional to support from the multidisciplinary assertive community treatment team.	7.5/10

Using the Risk and Vulnerability framework (See Table 4), we did not identify any key findings that highlight biomedical factors that influence the acceptability of health and structural interventions. We identified 11 key findings at the behavioural and structural levels of the Risk and Vulnerability framework that influence the acceptability of services; these findings are described below. The GRADE-CERQual confidence in findings ranged from very low to moderate (see Table 5). Confidence levels were downgraded due to methodological limitations, heterogeneity in populations and interventions, and the coherence of the data.

**Table 4:** Framework analysis key findings.

Framework level	Key Findings
Biomedical	None identified
Behavioural	**Self-identity:** Individuals who have lived on the street may have difficulty giving up the homeless identity. Many homeless individuals have difficulty adapting to their new environments and they undergo an adjustment period to become accustomed to the new structured community. Youth in particular felt that shelter environments did not foster the development of young adults, and they found it challenging to assimilate to their institutional nature. However, others recognized the importance of rules and structure. Illustrative quote: "*I felt vulnerable being there*” [68]
	**Autonomy**: Participants felt that having a sense of autonomy improved their motivation. However, some participants felt loss of autonomy in programs due to their perception of providers’ controlling attitudes. Illustrative quote: "We get here and we’re still treated like kids because some people don’t follow the rules; we can’t prove that we’re capable of managing our own matters. They don’t give us a chance to do it, so we’ll leave here without having that experience.” [79]
	**Pride:** Mothers expressed feelings of pride when they observed positive changes in their children’s health and social outcomes after their participation in maternal programs. They felt that family-oriented interventions allowed them to become effective mothers. Illustrative quote: “*It’s awesome because it makes me feel like I’m doing the right thing you know*, *that I’m being a good parent*” [54]
Structural	**Trust**: Homeless men and women of all ages value being able to trust their service providers and others around them. They felt that once trust was established, a strong relationship could develop. Male youth had more difficulty than other groups to trust providers and access services. Illustrative quote: "*My employment specialist made trusting so easy*. *She was quick to try to build confidence and trust*. *I was hesitant at first*, *but she really put me at ease*." [81]
	**Personal Safety:** Stable housing and service programmes create a sense of safety, security and stability for homeless individuals, who often face hardships in securing a safe place to sleep. Women-specific programmes create safe spaces for women to engage with each other in an environment free from violence and trauma. Such programs promote empowerment, self-efficacy and self-esteem. Illustrative quote: "… *when you have a roof it just doesn't matter*, *the rest doesn't matter*, *because you are in a safe place*. *[*.* *.* *.*] the most important thing was … that you were away from what you feared and … the life of … where you don't have to watch your back all the time*” [75]
	**Housing options:** People who are homeless or vulnerably housed often felt that the housing options available to them conflicted with their personal needs or were unattainable due to restrictive eligibility criteria. Youth expressed interest in congregate housing models, as these promote companionship, a sense of community, and a sense of family with others in similar circumstances. Women prioritized the safety of their children, and expressed concerns regarding the safety of the neighbourhoods in which they lived. Many women preferred integrated services within housing programs. Illustrative quote: "*No*. *I like the apartment*, *the apartment is great*. *This neighborhood*? *No*. *There’s been shootings for the last 4 days*, *loud music*, *people going in and out the building*, *it wakes the kids up*”. [82]
	**Continuity:** Many homeless individuals indicated challenges in maintaining adequate health when there were high turnover rates of service providers resulting in the discontinuity of care. The transient nature of worker support inhibited progress towards homeless persons’ goals and was detrimental to their ability to seek out and utilize services. Youth in particular highlighted the need for stability, continuity and commitment by support workers. Illustrative quote: "*My problem is they don’t have people that’s there on a regular basis*. *Like when I was brought in I had this one case manager*, *I was comfortable with her*, *and then I turn around and she’s leaving*.... *I need consistency*". [82]
	**Peer support:** Individuals participating in housing programs initially felt socially isolated, but found that support from peers and staff with shared lived experience of marginalization allowed them to develop altruism towards their peers. The key relationships identified were among staff, peers, friends, family, cultural traditions, and the community. Youth and women programs especially found that peer support helped develop relationships and enhanced commitment to seek treatment. Illustrative quote: “*I got somebody that’s there to help me*, *that’s supportive to me*, *that’s like a friend to me*, *I can always call them [staffs] and talk to them anytime of the day … She never mistreated me*, *talked about my business to other people*”. [82]
	**Empowerment**: Homeless and vulnerably housed individuals appreciated the pivotal role caseworkers played in providing them with tools to promote their empowerment and their independence. Quick connections to appropriate services may lead to rapid results, which some service users needed to see to sustain motivation to remain engaged. Illustrative quote: "*Case managers*, *I think*, *are very important for this program to be successful… I don’t think without a case manager*, *and having the follow up program*, *I don’t think many people would be successful*." [63]
	**Gender:** Socially constructed gender roles (assigned to men and women based on cultural beliefs, values, employment, and family role) may sometimes restrict access to programs. For example, men face difficulties being vulnerable and building trust, which inhibit their access to support services. Women experience restrictive relationships with men and family members, limiting their ability to take on roles other than the mother/caretaker. However, in one study, women felt that their family role acted as facilitators of positive program outcomes. Illustrative quote: “*Coming off the streets*, *[males] they’re not going to trust nobody except for themselves*”. [84]
	**Mental health:** Mental health problems and related stigma are exacerbated by the intergenerational cycle of violence and poverty experienced by vulnerably housed individuals. Youth feel particularly stigmatized for admitting to mental health issues, leading to an expectation of failure and low self-worth. As participants gain insights into their mental illness, they gain a sense of control over their own lives. Illustrative quote: “*It’s difficult for me to ask for help*.... *I don’t want to tell people that I have mental health issues or that I have a substance abuse problem*.... *They’re gonna think that I’m dirty*, *they’re gonna think I’m a prostitute*, *they’re gonna think that I use dirty needles*.” [69]

**Table 5:** CERQual summary of findings.

Review Finding	CERQual Assessment of Confidence in the Evidence	Explanation of CERQual Assessment	Studies Contributing to the Review Finding
**Stable housing and service programmes create a sense of safety, security and stability for homeless individuals, who often face hardships in securing a safe place to sleep. Women-specific programmes create safe spaces for women to engage with each other in an environment free from violence and trauma. Such programs promote empowerment, self-efficacy and self-esteem.** Illustrative quote: "… *when you have a roof it just doesn't matter*, *the rest doesn't matter*, *because you are in a safe place*. *[*.* *.* *.*] the most important thing was … that you were away from what you feared and … the life of … where you don't have to watch your back all the time*” [75]	Moderate confidence	While the majority of studies were consistent in reporting feelings of security, safety, and stability, one study contradicted this finding. Other concerns were about the generalizability of certain subgroups of people across the homeless population.	[26,56,63,67,68,72,73,75,76,79]
**Individuals who have lived on the street may have difficulty giving up the homeless identity. Many homeless individuals have difficulty adapting to their new environments and they undergo an adjustment period to become accustomed to the new structured community. Youth in particular felt that shelter environments did not foster the development of young adults, and they found it challenging to assimilate to their institutional nature. However, others recognized the importance of rules and structure.** Illustrative quote: "*I felt vulnerable being there*” [68]	Moderate confidence	Moderate concerns were about the generalizability of certain subgroups of people across the homeless population.	[56,67,68,72,77,79]
**People who are homeless or vulnerably housed often felt that the housing options available to them conflicted with their personal needs or were unattainable due to restrictive eligibility criteria.** **Youth expressed interest in congregate housing models, as these promote companionship, a sense of community, and a sense of family with others in similar circumstances.** **Women prioritized the safety of their children, and expressed concerns regarding the safety of the neighbourhoods in which they lived. Many women preferred integrated services within housing programs.** Illustrative quote: "*No*. *I like the apartment*, *the apartment is great*. *This neighborhood*? *No*. *There’s been shootings for the last 4 days*, *loud music*, *people going in and out the building*, *it wakes the kids up*”. [82]	Low confidence	Moderate concerns surrounding research design and small sample sizes. Additionally, studies often focused on narrow population groups, limiting their generalizability to all homeless populations.	[55,56,58,60,67,72,80,82,86]
**Individuals participating in housing programs initially felt socially isolated, but found that support from peers and staff with shared lived experience of marginalization allowed them to develop altruism towards their peers. The key relationships identified were among staff, peers, friends, family, cultural traditions, and the community.** **Youth and women programs especially found that peer support helped develop relationships and enhanced commitment to seek treatment.** Illustrative quote: “*I got somebody that’s there to help me*, *that’s supportive to me*, *that’s like a friend to me*, *I can always call them [staffs] and talk to them anytime of the day … She never mistreated me*, *talked about my business to other people*”. [82]	Moderate confidence	Moderate concerns were about the generalizability of certain subgroups of people across the homeless population and the variability in interventions administered	[26,54,56,58,60,62,64,66,69,72,73,76,84,86,87]
**Mental health problems and related stigma are exacerbated by the intergenerational cycle of violence and poverty experienced by vulnerably housed individuals.** **Youth feel particularly stigmatized for admitting to mental health issues, leading to an expectation of failure and low self-worth. As participants gain insights into their mental illness, they gain a sense of control over their own lives.** Illustrative quote: “*It’s difficult for me to ask for help*.... *I don’t want to tell people that I have mental health issues or that I have a substance abuse problem*.... *They’re gonna think that I’m dirty*, *they’re gonna think I’m a prostitute*, *they’re gonna think that I use dirty needles*.” [69]	Very low confidence	Moderate concerns around methodological limitations, such as limited justification of the research design and analysis, and some studies did not consider ethical issues. Additionally, certain subgroups were emphasized, challenging the applicability of results to the larger public, and some studies did not have supporting evidence for the key findings	[54,56,59,61,66,67,69–72,75,77,81,82,84–86]
**Homeless men and women of all ages value being able to trust their service providers and others around them. They felt that once trust was established, a strong relationship could develop.** **Male youth had more difficulty than other groups to trust providers and access services.** Illustrative quote: "*My employment specialist made trusting so easy*. *She was quick to try to build confidence and trust*. *I was hesitant at first*, *but she really put me at ease*." [81]	Moderate confidence	Moderate concerns surrounding methodological limitations such as limited justification of research design and analysis and small sample sizes.	[26,57,61,64,68,70,72,79–82,84–86]
**Participants felt that having a sense of autonomy improved their motivation. However, some participants felt loss of autonomy in programs due to their perception of providers’ patronizing attitudes.** Illustrative quote: "*We get here and we’re still treated like kids because some people don’t follow the rules; we can’t prove that we’re capable of managing our own matters*. *They don’t give us a chance to do it*, *so we’ll leave here without having that experience*.” [79]	Moderate confidence	Moderate concerns were raised with respect to methodological limitations. Only minor concerns were referenced with respect to relevance and adequacy due to small sample sizes and poor indication of the number of participants who supported a claim, respectively	[65,72,79]
**Homeless and vulnerably housed individuals appreciated the pivotal role caseworkers played in providing them with tools to promote their empowerment and their independence. Quick connections to appropriate services may lead to rapid results, which some service users needed to see to sustain motivation to remain engaged.** Illustrative quote: "*Case managers*, *I think*, *are very important for this program to be successful… I don’t think without a case manager*, *and having the follow up program*, *I don’t think many people would be successful*." [63]	Very low confidence	Major concerns were attributed to the coherence of the data, as two studies commented on the potential harms caused by caseworkers and service providers. Additionally, variation in sampled program participants may influence generalizability to homeless populations.	[58,62,63,67,68,70,75,76,82,86]
**Many homeless individuals indicated challenges in maintaining adequate health when there were high turnover rates of service providers resulting in the discontinuity of care.** **The transient nature of worker support inhibited progress towards homeless persons’ goals and was detrimental to their ability to seek out and utilize services.** **Youth in particular highlighted the need for stability, continuity and commitment by support workers.** Illustrative quote: "*My problem is they don’t have people that’s there on a regular basis*. *Like when I was brought in I had this one case manager*, *I was comfortable with her*, *and then I turn around and she’s leaving*.... *I need consistency*". [82]	Low confidence	Moderate concerns were raised with regards to the generalizability of study findings due to the focus on specific subgroups (ie. HIV+ mothers). While the key findings were generally well supported, one article provided limited supporting evidence	[55,56,58,62,64,66–68,70,72,77,79,80,82,86]
**Socially constructed gender roles (assigned to men and women based on cultural beliefs, values, employment, and family role) may sometimes restrict access to programs.** **For example, men face difficulties being vulnerable and building trust, which inhibit their access to support services.** **Women experience restrictive relationships with men and family members, limiting their ability to take on roles other than the mother/caretaker.** **However, in one study, women felt that their family role acted as facilitators of positive program outcomes.** Illustrative quote: “*Coming off the streets*, *[males] they’re not going to trust nobody except for themselves*” [84]	Moderate confidence	Moderate concerns were about the generalizability of certain subgroups of people across the homeless population and the relatively small sample sizes in studies	[65,66,74,75,84]
**Mothers expressed feelings of pride when they observed positive changes in their children’s health and social outcomes after their participation in maternal programs. They felt that family-oriented interventions allowed them to become effective mothers.** Illustrative quote: “*It’s awesome because it makes me feel like I’m doing the right thing you know*, *that I’m being a good parent*” [54]	Low confidence	Significant concerns were raised with regards to the generalizability of study findings due to small sample sizes and the focus on specific subgroups (ie. African Americans, HIV+ mothers). One study contradicted the findings relevant to the key finding, and while the key finding was generally well supported, one article only have one supporting piece of evidence	[54,66,71,82]

We identified three key findings that influence intervention acceptability at the behavioural level:

### Self-identity

Studies found that one’s sense of self may be challenged as they adapt to a life off the streets. Individuals with a history of homelessness found it challenging to give up the homeless identity/community. Many individuals who experienced homelessness had difficulty adapting to new environments and underwent an adjustment period to become accustomed to new structured communities and lifestyles,[68,72,77,79] for example, requiring several months to feel worthy of “walking on the sidewalks instead of in the back alleys.”[72] Youth, in particular, felt that shelter environments did not foster the development of young adults and found it particularly challenging to assimilate to the shelters’ institutional nature.[56] Others recognized the importance of rules and structure as essential elements for personal development.[67]

### Autonomy

Participants felt that having a sense of autonomy while participating in programs improved their motivation to “do what it takes” to maintain positive outcomes. However, some participants felt a loss of autonomy when involved in programs due to their perception of providers’ patronizing attitudes.[65,72,79] For example, participants reported being “treated like kids”[79] and being “told what to do”.[65]

### Pride

Mothers expressed feelings of pride when they observed positive changes in their children’s health and social outcomes after participating in maternal programs. They felt that family-oriented interventions allowed them to become effective mothers [54,66,71,82]: “It’s awesome because it makes me feel like I’m doing the right thing you know, that I’m being a good parent”.[54]

We identified eight key findings that influence intervention acceptability and accessibility at the structural level:

### Personal safety

Stable housing and service programs created a sense of safety, security and stability for individuals with lived experience of homelessness, who often faced hardships in securing safe places for personal belongings and activities [26,56,63,67,68,72,73,76,79,85], “where you don’t have to watch your back all the time” [75]. Women, in particular, found that women-specific programs created safe spaces to engage with one another in an environment free from violence and trauma. Such programs promoted feelings of empowerment, self-efficacy, and enhanced self-esteem.[74,75]

### Housing options

People who experienced homelessness or vulnerable housing often felt that the limited housing options did not address their personal needs and preferences, such as preferred neighbourhoods or housing with sufficient privacy, or were unattainable due to restrictive eligibility criteria.[55,56,58,60,72,80,86] Youth expressed interest in congregate housing models, as these promoted companionship and a sense of community and surrogate family with others in similar circumstances.[67,86] Women prioritized the safety of their children and expressed concerns regarding the safety of the neighbourhoods in which they lived.[72,82] Many women communicated a preference for integrated services within housing programs.[82]

### Trust

Men and women of all ages with lived experience of homelessness valued being able to trust their service providers and others around them. They felt that once trust was established, a strong relationship could develop, and could facilitate relationship-building for others: “[…] as soon as a couple of them start to trust you, you start to get everyone else in the hostel to know you”.[57] However, male youth had more difficulty trusting providers and accessing services than other groups. [26,57,58,61,64,68,72,79–82,84–86]

### Empowerment

Once trust was established, persons who experienced homelessness or vulnerable housing appreciated the pivotal role caseworkers played in providing them with tools to promote their empowerment and independence.[58,62,63,67,68,70,75,82,86] Quick connections to appropriate services may lead to rapid results, which some service users needed to see to sustain motivation to remain engaged.

### Continuity of care

Many persons who experienced homelessness identified challenges in accessing care and maintaining adequate health when there were high turnover rates of service providers, thus resulting in a discontinuity of care. The transient nature of providers was detrimental to the ability of persons experiencing homelessness to seek out and utilize services. One participant said “Sometimes you need something solid in your life. Not knowing, especially for an addict… the unknown brings fear. It ends after three years. […]. it worries me a lot, the ‘after’ here”[79]. Youth, in particular, highlighted the need for stability, continuity and commitment by support workers. [55,56,58,62,64,66,70,77,79,80,82,86]

### Peer support

Individuals participating in housing programs initially felt socially isolated but found that support from peers and staff with shared lived experience of marginalization allowed them to develop strong connections with and eventual feelings of altruism toward their peers.[58,60,62,67,68,72,76,84] The key relationships identified were with staff,[62,66,72,73,86] peers, [56,64,67,69,84] friends,[64] family,[54,66,69] people from their cultural community,[60,72] and the broader community.[67,72] These individuals also found that the peer support in youth- and women-specific programs was especially beneficial to the development of relationships and in enhancing one’s commitment to seek treatment and participate in programs.[26,54,56,64,67,69,73,84,87]

### Gender constructs

Socially constructed gender roles (assigned to men and women, based on cultural beliefs, values, employment, and family role) were found to result in restricted access to programs.[65,66,74,75,84] For example, men faced difficulties in showing their vulnerability and in building trust, which inhibited their openness to accessing support services. Women reported restrictive relationships with men and family members, which limited their ability to take on roles other than as the mother/caretaker. However, in one study, women felt that their family role facilitated positive program outcomes.[74]

### Mental health

Finally, studies highlighted that the intergenerational cycle of violence and poverty experienced by individuals who are vulnerably housed exacerbated their mental health problems and related stigma.[66,67,70] This was particularly prominent among women [54,75,82] and their children.[71] Youth, in particular, felt stigmatized for admitting to having mental health issues; this led to feelings of low self-worth and expectations of failure.[59,69,72,77,81,84] As participants gained insights into their mental illness, they acquired a sense of control over their own lives.[56,61,67,72,85,86]

## Discussion

We found several behavioural and structural factors that influenced the acceptability of health and structural interventions, including permanent supportive housing, case management, income, interventions for substance use, and women- and youth-focused interventions, for persons who are homeless or vulnerably housed. We found that several behavioural and structural factors influenced the acceptability of these interventions. The included studies reported, with moderate confidence, that persons with lived experience of homelessness described recurrent experiences of marginalization, dehumanization, and exclusion, and that they highly valued trust and safety within human interactions. Other key findings with moderate confidence included the need for autonomy and support in relationships and that gender should be considered in intervention delivery. Trust and safety and the negative impacts of structural violence resonated across the studies. As the paradigm of safety expands to all settings within the healthcare continuum,[88] there is a need for anti-oppressive approaches (critically reflecting on the power structures such as systemic racism, sexism, homophobia, transphobia, ableism that impact patients’ lives) [89,90] that address structural violence. Primary care providers are uniquely positioned to provide, refer to, or advocate for health and structural interventions using the principles of trauma-informed care [91]

Trauma-informed care involves the following five principles: trauma awareness and acknowledgment; safety and trustworthiness; choice, control, and collaboration; strengths-based and skills-building care; and cultural, historical, and gender issues. The establishment of trust allows for the development of self-identity and small daily interactions that contribute to broader social connections.[92–94] Furthermore, trust in healthcare practitioners improves the likelihood of persons experiencing homelessness or vulnerable housing seeking assistance and adhering to treatment.[95] Individuals who experienced homelessness and vulnerable housing preferred a continuity of services and a consistent/stable group of service providers/practitioners.[55,71] Additionally, supporting empowerment, [91] resilience and a sense of safety develops over time,[92] suggesting an important function for longitudinal strengths-based or skill-building roles, such as case management and peer support. Peers were a preferred avenue for support, given that persons with a shared lived experience of homelessness may understand each other and the challenges they face in navigating fragmented health and social systems. Persons with lived experience of homelessness valued a physically and emotionally safe setting for interventions. For example, mothers emphasized personal safety when securing adequate housing and services for themselves and for their children. Results from this review also showed that persons with lived experience valued having integrated services or a “one-stop shop” situation where various needs could be met and services could be coordinated.

Our review also shows that persons with lived experience of homelessness undergo a shift in self-identity. Primary care providers should remain open to personal and social identity and marginalization.[91] Many individuals who had experienced homelessness underwent an adjustment period to become accustomed to new structured communities and lifestyles.[68,72] A new living environment and community allowed for the reconfiguring of one’s identity.[72] In general, participants who maintained positive self-identities while homeless related more strongly to street culture and related peers.[72] The process of acculturation may have an impact on both their social and psychological well-being and can also promote health by creating access to previously inaccessible health and social services.[96] As persons with lived experience undergo a shift in their cultural values and beliefs, their self-perception, such as having competent parenting abilities, contributes to a positive self-identity; as a caregiver for example. Mothers who experienced homelessness and vulnerable housing expressed pride when they observed their children's’ positive changes in health and social outcomes, due to their participation in maternal programs.[54,66,71,82] Throughout our findings, pride was identified as an emotional attribute that was linked to pro-social behaviour.

Our results underscore the necessity of there being an intersectional approach that considers age and gender when developing interventions and services for people who experience homelessness.[97,98] Men and women have different experiences of homelessness,[99–101] and these should be reflected in service provision and evaluation.[102,103] For instance, previous research has shown that homelessness or unstable housing among women can be harder to detect due to hesitancy in self-identifying as such due to the associated stigma.[104] Furthermore, trauma or severed social ties upon separation from an abusive partner can increase the risk of social isolation and emotional distress. Including equity-related considerations around sex, gender and diversity can increase the reach and impact of interventions; thus, enabling these interventions to better meet the diverse needs of homeless women and their families.

Similarly, age should also be considered when developing and delivering interventions for persons with lived experience of homelessness.[105] For example, older adults who experience homelessness face challenges in terms of the continuity of case management and care,[80] while youth face the challenge of breaking the cycle of generational poverty and homelessness. Key challenges faced by youth include social isolation, alienation, low self-worth, lack of resources, and substance abuse.[84] Young people are drawn to providers who convey openness and acceptance, emphasize establishing trust, and instill hope.[86] The physical environment of shelters and housing services represents a structural barrier for youth, as they find it difficult to assimilate to the institutional nature of the shelter environment.[56] Furthermore, youth expressed a need for life-skills services, such as education, managing finances and obtaining housing, which would allow them to establish autonomy and meet their personal goals.

This systematic review offers a pragmatic response to time-critical projects [47] and the GRADE-CERQual approach assesses the confidence levels of the key findings. However, using secondary data is limited by the information provided in published primary studies. Furthermore, employing the ‘best-fit’ variant of framework analyses requires assurance that data is not forced into the *a priori* framework; rather new themes are created as needed. Using secondary data and ‘best fit’ strategies may be very helpful when policymakers, practitioners or other decision makers need answers quickly, and are able to tolerate some ambiguity about whether the answer is the very best that could be given [49]. The literature included in this qualitative analysis was restricted to seven intervention groups that were identified through a modified Delphi consensus process by both persons with lived experience of homelessness and recognized experts in the field; however, this approach may have missed relevant findings from studies examining other interventions. We also recognize the possibility that valuable insights may have been excluded from the synthesis through the exclusion of low-quality studies, an assessment process which is itself subjective and relies on varying levels of reporting standards. Finally, the exclusion of indigenous-specific interventions limits the applicability of these findings to this population.

The experiences among individuals who are homeless varies greatly; therefore, including persons with lived experience of homelessness in the development and implementation of interventions may help ensure that the needs of the community are met and will facilitate their sense of choice and control over their care. Incorporating their values and preferences is necessary to create appropriate and effective programs and interventions with greater chances of success in serving this population and in reducing health inequities. Key factors to be considered in the development of new programs include the behavioural and structural influences that dictate service acquisition and adherence. Current and future primary care and public health interventions should be developed and implemented with particular attention paid to the factors relevant to transitions in care identified in this review, such as trust, personal safety, and the continuity of care. However, it should be noted that there exists challenges in commissioning services, instituting changes in service provision, and the development of specific care pathways for persons experiencing homelessness when public services are subject to austerity measures. This review’s findings contribute to biomedical and healthcare strategies (e.g., harm-reduction strategies); educational and behavioural strategies (e.g., counselling and referral services); environmental strategies that support and facilitate behaviour change; and policy and legislative strategies (e.g., related to poverty, social housing, law enforcement) [106]. Establishing a trusting relationship at first contact with service providers and primary care practitioners is the foundation for positive outcomes.

## Supporting information

S1 File PRISMA checklist. (DOCX)

S2 File Intervention descriptions. (PDF)

S3 File Inclusion and exclusion criteria. (PDF)

S4 File Search strategy. (PDF)

S5 File Grey literature search. (PDF)

S6 File CASP assessments. (PDF)

S7 File List of excluded studies. (PDF)
