# Supplementary material for: Common trust and personal safety issues: A systematic review on the acceptability of health and social interventions for persons with lived experience of homelessness
Source: PLoS One. 2019 Dec 30;14(12):e0226306. doi: 10.1371/journal.pone.0226306 (PMC6936789; doi:10.1371/journal.pone.0226306)
Supplement: S2 File — (PDF) [file pone.0226306.s002.pdf]

Appendix II: Description of Interventions

Category	Intervention	Description	How were these interventions selected?
**Housing interventions**	Housing First/permanent supportive housing	An evidence-based supportive housing intervention for homeless populations experiencing mental illness and substance use	We conducted a Delphi consensus process with 84 practitioners and 76 persons with lived experience of homelessness to select priority intervention categories and populations (See Shoemaker et al., submitted). We established Expert Working Groups for each category of interventions comprising practitioners, researchers and persons with lived experience of homelessness. We conducted scoping exercises to identify specific interventions within each category and selected interventions of interest by consensus.
**Mental health interventions**	Assertive community treatment (ACT)	Offers team-based care by a multidisciplinary team of healthcare workers that provide services tailored to the needs of each person	
	Intensive case management (ICM)	Helps service users maintain housing and achieve a better quality of life through the support of a case manager	
	Pharmacological interventions for psychosis	Effectiveness of injectable antipsychotics as a first line of treatment for homeless individuals in precarious situations	
**Substance use interventions**	Supervised consumption facilities	Legally sanctioned facilities where people who use substances can consume pre-obtained substances under supervision	
	Managed alcohol programs	Includes shelter, medical assistance, social services and the provision of regulated alcohol to help residents cope with alcohol dependence.	
	Pharmacological interventions for opioid use disorder	Opioid therapy medications including methadone, buprenorphine, diacetylmorphine, levo-α-acetylmethadol (LAAM) and naltrexone	
	Pharmacological agents for reversal of opioid overdose	Opioid antagonist administered intravenously or intranasally; e.g., naloxone	
**Interventions for care coordination and case management**	Peer support	Provision of encouragement, affiliation and services by or with an individual who has experienced a similar background to the service user	
	Non-intensive case management	Including clinical case management and standard case	

		management that allow for the provision of an array of social, healthcare, and other services with the goal of helping individuals maintain good health and strong social relationships	
**Interventions for income assistance**	Direct income assistance	Benefits and programs offered by individuals or institutions that increase income with the goal of improving socioeconomic status	
	Cost reduction support/indirect income assistance	Addresses critical social determinants of health needs for which a person would otherwise be paying out of their basic income	
**Interventions for women and youth**	Women	Motivational interview counselling, structured education sessions, therapeutic communities, and multimodal interventions	
	Youth	Place-based interventions, youth- and family-focused therapy interventions, parental monitoring interventions, and street outreach and addictions services	
