# Supplementary material for: Common trust and personal safety issues: A systematic review on the acceptability of health and social interventions for persons with lived experience of homelessness
Source: PLoS One. 2019 Dec 30;14(12):e0226306. doi: 10.1371/journal.pone.0226306 (PMC6936789; doi:10.1371/journal.pone.0226306)
Supplement: S3 File — (PDF) [file pone.0226306.s003.pdf]

Appendix III: Inclusion/Exclusion Criteria

**Population**	We will consider studies of homeless and vulnerably housed individuals of high-income countries.
**Setting**	Health and social-service settings in high-income countries. Studies conducted in low- and middle-income countries will be excluded. No restriction on rural or urban settings.
**Interventions**	Interventions of interest include interventions for housing, care coordination, income, mental health and addiction, and women and youth described above. However, studies will be included in our review as long as the intervention is related and/or generalizable to the six topics of interest.
**Comparison**	No intervention or other intervention comparison.
**Outcomes**	Identification of the factors that affect patient perspectives, views, attitudes and beliefs regarding the intervention(s) and the barriers and enabling factors to implementation and use; valuation of positive and negative intervention outcomes; views about the acceptability and accessibility of interventions.
**Study Design**	The review will focus on the values and perceptions of homeless and vulnerably housed persons, which is most appropriately answered through qualitative research. Qualitative methods may include, but are not limited to, ethnography, grounded theory or phenomenology. Any study that utilizes survey data or statistical reporting of results will be excluded, as will commentaries or discussions on the subject. Qualitative data from a mixed-methods study will be excluded.
**Restrictions**	Date of publication is limited to the period January 1994 to January 2018. No language restrictions.
