# Supplementary material for: Common trust and personal safety issues: A systematic review on the acceptability of health and social interventions for persons with lived experience of homelessness
Source: PLoS One. 2019 Dec 30;14(12):e0226306. doi: 10.1371/journal.pone.0226306 (PMC6936789; doi:10.1371/journal.pone.0226306)
Supplement: S4 File — (PDF) [file pone.0226306.s004.pdf]

Appendix IV: Search Strategy

1 exp homeless persons/ or exp homeless man/ or exp homeless youth/ or exp homeless woman/

2 (homeless* or underhouse* or roofless* or unhouse* or squatter* or shelter* or unsheltered).ti,ab.

3 ("no fixed address" or "seeking shelter" or "street involved" or "sleeping rough" or "unstable housing" or "housing instability" or "precarious housing" or "precariously housed" or "lack of housing" or "rough sleep" or "vulnerably housed").ti,ab.

4 (((homeless or street or transient* or marginal* or vulnerabl*) adj2 (population or person or persons or people* or individual or individuals or adult or adults or child* or youth* or men or man or women or woman)) or ((temporary or unstabl* or vulnerabl*) adj2 (hous* or accommodation* or shelter* or hostel* or dwelling*))).ti,ab.

5 1 or 2 or 3 or 4

6 exp health knowledge, attitudes, practice/ or exp patient satisfaction/ or exp patient preference/ or exp health services accessibility/ or exp health equity/ or exp Attitude to Health/ or exp self-efficacy/ or exp Adaptation, Psychological/ or exp health education/ or exp Health Risk Behaviors/ or exp social behavior/

7 (patient adj3 (value* or preference* or belie* or attitude? or perspective* or view*)).ti,ab.

8 ((access or accessib*) adj5 (care or health*)).tw.

9 (acceptable or acceptabilit$ or prefer$ or satisf$ or useful$ or utility or value$ or perspective* or view* or perceived or belie* or knowledge or expect*).ti,ab.

10 ((biomedic* or behavio?r* or structur* or physical or environment* or social or politic* or econom* or cultur*) adj5 (factor* or barrier* or facilitator*)).ti,ab.

11 6 or 7 or 8 or 9 or 10

12 ((("semi-structured" or semistructured or unstructured or informal or "in-depth" or indepth or "face-to-face" or structured or guide) adj3 (interview* or discussion* or questionnaire*)) or (focus group* or qualitative or ethnograph* or fieldwork or "field work" or "key informant")).ti,ab. or interviews as topic/ or focus groups/ or narration/ or qualitative research/

13 5 and 11 and 12

14 limit 13 to yr="1994 -Current"
