# Supplementary material for: Common trust and personal safety issues: A systematic review on the acceptability of health and social interventions for persons with lived experience of homelessness
Source: PLoS One. 2019 Dec 30;14(12):e0226306. doi: 10.1371/journal.pone.0226306 (PMC6936789; doi:10.1371/journal.pone.0226306)
Supplement: S5 File — (PDF) [file pone.0226306.s005.pdf]

Appendix V: Grey Literature Search

<table>
<tr><th>Source consulted</th><th>Search terms</th></tr>
<tr><td>Homeless Hub</td><td rowspan="5">1. “Homeless” AND
2. “Housing First” OR “Mental Health” OR “Case Management” OR “Addictions” OR “Harm Reduction” OR “Income” AND
3. “Acceptance” OR “Access” OR “Acceptability” OR “Accessibility” OR “Qualitative”

Population-specific terms: “Women” OR “Mother” OR “Children” OR “Youth” OR “Disability”</td></tr>
<tr><td>Health Canada</td></tr>
<tr><td>Public Health Agency of Canada</td></tr>
<tr><td>Canadian Public Health Association</td></tr>
<tr><td>Canadian Mortgage and Housing Corporation</td></tr>
</table>
