# Supplementary material for: Common trust and personal safety issues: A systematic review on the acceptability of health and social interventions for persons with lived experience of homelessness
Source: PLoS One. 2019 Dec 30;14(12):e0226306. doi: 10.1371/journal.pone.0226306 (PMC6936789; doi:10.1371/journal.pone.0226306)
Supplement: S6 File — (PDF) [file pone.0226306.s006.pdf]

Appendix S6: CASP assessments

Study ID	Was there a clear statement of the aims of the research?	Is a qualitative methodology appropriate?	Was the research design appropriate to address the aims of the research?	Was the recruitment strategy appropriate to the aims of the research?	Was the data collected in a way that addressed the research issue?	Has the relationship between researchers and participants been adequately considered?	Have ethical issues been taken into consideratio n?	Was the data analysis sufficiently rigorous?	Is there a clear statement of findings?	How valuable is the research?	Total score
Alhusen et al. 2017	Yes	Yes	Yes	Can't tell	Yes	Can't tell	Yes	Yes	Yes	Yes	9
Archard & Murphy 2015	Yes	Yes	No	Yes	Yes	No	Yes	Can't tell	Yes	Yes	7.5
Aviles & Helfrich 2004	Yes	Yes	Can't tell	Yes	Yes	No	Can't tell	Yes	Yes	Can't tell	7.5
Barker et al. 2018	Yes	Yes	Yes	Can't tell	Yes	Can't tell	Yes	Yes	Yes	Yes	9
Canham et al. 2019	Yes	Yes	Yes	Yes	Yes	No	Yes	Yes	Yes	Yes	9
Chaturvedi 2016	Yes	Yes	Yes	Yes	Yes	Yes	Yes	Yes	Yes	Yes	10
Collins et al. 2012	Yes	Yes	Yes	Can't tell	Yes	No	Can't tell	Yes	Yes	Yes	8
Cormack 2009	Yes	Yes	Can't tell	Yes	Yes	Yes	Yes	Yes	Can't tell	Yes	9
Davis et al. 2012	Yes	Yes	Yes	Yes	Yes	Yes	Yes	Yes	Yes	Yes	10
Farquhar et al. 2014	Yes	Yes	Can't tell	Yes	Yes	Yes	Yes	Yes	Yes	Yes	9.5
Ferguson & Islam 2008	Yes	Yes	Yes	Yes	Yes	Yes	Yes	Yes	Yes	Yes	10
Guilcher et al. 2016	Yes	Yes	Yes	Yes	Yes	No	Yes	Yes	Yes	Yes	9

Gultekin et al. 2014	Yes	Yes	Yes	Yes	Yes	Yes	Yes	Yes	Yes	Can't tell	9.5
Holtschneider 2016	Yes	Yes	Yes	Yes	Yes	No	Can't tell	Yes	Yes	Yes	8.5
Jost et al. 2011	Yes	Yes	Yes	Yes	Yes	Yes	Yes	Yes	Yes	Yes	10
Kozloff et al. 2013	Yes	Yes	Yes	Yes	Yes	Can't tell	Yes	Yes	Yes	Yes	9.5
Lamanaa et al. 2018	Yes	Yes	Yes	Yes	Yes	Can't tell	Yes	Yes	Yes	Yes	9.5
Lorelle & Grothaus 2015	Yes	Yes	Yes	Yes	Yes	Yes	Yes	Yes	Yes	Yes	10
Macnaughton et al. 2016	Yes	Yes	Yes	Yes	Can't tell	No	No	Yes	Yes	Yes	7.5
Macneil & Pauly 2011	Yes	Yes	Yes	Can't tell	Yes	No	Yes	Can't tell	Yes	Yes	8
Magee et al. 2008	Yes	Yes	Yes	Can't tell	Yes	No	Yes	Yes	Yes	Yes	8.5
McMaster et al. 2017	Yes	Yes	No	Can't tell	Yes	No	Yes	Yes	Yes	Yes	7.5
Mitchell et al. 2017	Yes	Yes	Yes	Yes	Yes	Can't tell	Yes	Yes	Yes	Yes	9.5
Patterson et al. 2015	Yes	Yes	Yes	Yes	Yes	No	Yes	Yes	Yes	Yes	9
Patterson et al. 2013	Yes	Yes	Can't tell	Yes	Can't tell	No	Yes	Yes	Yes	Yes	8
Perreault et al. 2016	Yes	Yes	No	Yes	Yes	No	Yes	Yes	Yes	Yes	8
Ploeg et al. 2008	Yes	Yes	Yes	Yes	Can't tell	No	Yes	Yes	Yes	Yes	8.5

Poremski et al. 2016	Yes	Yes	No	Yes	Can't tell	No	Yes	Yes	Yes	Yes	7.5
Quinn et al. 2015	Yes	Yes	Yes	Yes	Yes	No	Yes	Yes	Yes	Yes	9
Radey & Wilkins 2010	Yes	Yes	No	Can't tell	Yes	No	Yes	Yes	Yes	Yes	7.5
Stewart et al. 2010	Yes	Yes	Yes	Yes	Yes	No	Yes	Yes	Yes	Yes	9
Taylor et al. 2007	Yes	Yes	Yes	Can't tell	No	Yes	Yes	Yes	Yes	Yes	8.5
Thompson et al. 2006	Yes	Yes	Yes	Yes	Yes	No	Can't tell	Yes	Yes	Yes	8.5
Wright et al. 2006	Yes	Yes	No	Yes	Yes	Can't tell	Yes	Yes	Yes	Yes	8.5
Yamin et al. 2014	Yes	Yes	No	Can't tell	Yes	No	Yes	Yes	Yes	Yes	7.5

The studies were assessed using a score from 1 to 10, with 10 being of highest quality and 1 the lowest. Studies were awarded 1 point for each "yes", 0.5 points for "can't tell" and 0 points for "no" (Butler et al., 2016). We excluded studies that scored below the 7.5 quality threshold. The mean CASP score for our included studies was 8.71 (SD 0.83).
