# Supplementary material for: Common trust and personal safety issues: A systematic review on the acceptability of health and social interventions for persons with lived experience of homelessness
Source: PLoS One. 2019 Dec 30;14(12):e0226306. doi: 10.1371/journal.pone.0226306 (PMC6936789; doi:10.1371/journal.pone.0226306)
Supplement: S7 File — (PDF) [file pone.0226306.s007.pdf]

Appendix VIII – Table of Excluded Studies

#	Qualitative Studies	Reason for exclusion
1	Christian J, Abrams D. A tale of two cities: Predicting homeless people's uptake of outreach programs in London and New York. Basic and Applied Social Psychology. 2004 Sep 1;26(2-3):169-82.	Wrong study design
2	Amen MM, Pacquiao DF. Contrasting experiences with child health care services by mothers and professional caregivers in transitional housing. Journal of Transcultural Nursing. 2004 Jul;15(3):217-24.	Wrong intervention
3	Anderson L, Stuttaford M, Vostanis P. A family support service for homeless children and parents: User and staff perspectives. Child & Family Social Work. 2006 May;11(2):119-27.	Low Quality
4	Applewhite SL. Homeless veterans: Perspectives on social services use. Social Work. 1997 Jan 1;42(1):19-30.	Wrong intervention
5	Applewhite SL. Coping and survival skills of homeless veterans. Journal of Applied Social Sciences. 1998;23:13-22.	Wrong intervention
6	August M. Revitalisation gone wrong: Mixed-income public housing redevelopment in Toronto's Don Mount Court. Urban Studies. 2016 Dec;53(16):3405-22.	Wrong intervention
7	Baird AF, Campanaro CS, Eisele JL, Hall T, Wright JD. "How Can We Stay Sober?" Homeless Women's Experience in a Substance Abuse Treatment Center. Society. 2014 Aug 1;51(4):404-7.	Low Quality
8	Beharry M. Deserving and Designing Health Care: Suggestions for Health Care Improvement From Homeless Teens. In2012 AAP National Conference and Exhibition 2012 Oct 19. American Academy of Pediatrics.	Wrong publication
9	Bender K, Barman-Adhikari A, DeChants J, Haffejee B, Anyon Y, Begun S, Portillo A, Dunn K. Asking for Change: Feasibility, acceptability, and preliminary outcomes of a manualized photovoice intervention with youth experiencing homelessness. Children and Youth Services Review. 2017 Oct 1;81:379-89.	Wrong study design
10	Biederman DJ. Understanding the experience of interacting with service providers from the perspective of homeless women: A phenomenological study. The University of North Carolina at Greensboro; 2012.	Wrong publication
11	Borysow ID, Furtado JP. Access, equity and social cohesion: evaluation of intersectoral strategies for people experiencing homelessness. Revista da Escola de Enfermagem da USP. 2014 Dec;48(6):1069-76.	Wrong geographical setting
12	Bowen EA, Miller B, Barman-Adhikari A, Fallin K, Zuchlewski D. Emerging adult homelessness in geographic perspective: A view from the Rust Belt. Children and Youth Services Review. 2017 Feb 1;73:213-9.	Wrong intervention
13	Brown H, Howlett F. A critical evaluation of the "short stay project"–service users' perspectives. Housing, Care and Support. 2017 Jun 5;20(2):71-84.	Wrong population
14	Brown M, Mihelicova M, Lyons J, DeFonzo J, Torello S, Carrión A, Ponce AN. Waiting for shelter: Perspectives on a homeless shelter's procedures. Journal of Community Psychology. 2017 Sep;45(7):846-58.	Wrong intervention
15	Brown SL. The history of housing and treatment services for people with serious psychiatric disabilities: Models of residential service delivery. University of Hartford; 2005.	Wrong publication
16	Campbell DJ, Gibson K, O'Neill BG, Thurston WE. The role of a student-run clinic in providing primary care for Calgary's homeless populations: a qualitative study. BMC health services research. 2013 Dec;13(1):277.	Wrong population
17	Carton AD, Young MS, Kelly KM. Changes in sources and perceived quality of social supports among formerly homeless persons receiving assertive community treatment services. Community Mental Health Journal. 2010 Apr 1;46(2):156-63.	Wrong study design

18	Caton S, Greenhalgh F, Goodacre L. Evaluation of a community dental service for homeless and'hard to reach'people. British dental journal. 2016 Jan;220(2):67.	Wrong intervention
19	Clifasefi SL, Collins SE. Community Based Participatory Research On Alcohol Harm Reduction Interventions Within Housing First Settings: 0229. Alcoholism: Clinical & Experimental Research. 2014 Jun 1;38:58A.	Wrong publication
20	Clifasefi SL, Collins SE, Torres NI, Grazioli VS, Mackelprang JL. HOUSING FIRST, BUT WHAT COMES SECOND? A QUALITATIVE STUDY OF RESIDENT, STAFF AND MANAGEMENT PERSPECTIVES ON SINGLE-SITE HOUSING FIRST PROGRAM ENHANCEMENT. Journal of community psychology. 2016 Sep;44(7):845-55.	Low Quality
21	Collins P, Barker C. Psychological help-seeking in homeless adolescents. International Journal of Social Psychiatry. 2009 Jul;55(4):372-84.	Wrong intervention
22	Dashora P, Slesnick N, Erdem G. "UNDERSTAND MY SIDE, MY SITUATION, AND MY STORY:" INSIGHTS INTO THE SERVICE NEEDS AMONG SUBSTANCE-ABUSING HOMELESS MOTHERS. Journal of community psychology. 2012 Nov;40(8):938-50.	Wrong intervention
23	Deverteuil G. The relationship between government assistance and housing outcomes among extremely low-income individuals: A qualitative inquiry in Los Angeles. Housing Studies. 2005 May 1;20(3):383-99.	Wrong study design
24	Doroshenko A, Hatchette J, Halperin SA, MacDonald NE, Graham JE. Challenges to immunization: the experiences of homeless youth. BMC public health. 2012 Dec;12(1):338.	Wrong intervention
25	Draucker CB, Johnson DM, Johnson-Quay NL, Kadeba MT, Mazurczyk J, Zlotnick C. Rapid HIV testing and counseling for residents in domestic violence shelters. Women & health. 2015 Apr 3;55(3):334-52.	Wrong intervention
26	Eccles DR. A phenomenological qualitative study of homeless individuals accessing health care (Doctoral dissertation, University of Phoenix).	Wrong publication
27	Elder NC, Tubb MR. Diabetes in homeless persons: barriers and enablers to health as perceived by patients, medical, and social service providers. Social work in public health. 2014 Apr 16;29(3):220-31.	Wrong intervention
28	Evans J, Semogas D, Smalley JG, Lohfeld L. "This place has given me a reason to care": understanding 'managed alcohol programs' as enabling places in Canada. Health & place. 2015 May 1;33:118-24.	Low Quality
29	Forchuk C, Giustizia S, Annett N, Connoy M, Csiernik R, Diaz K, Edwards B, Elkin D, Fisman S, Godin M, Jeng M. FC27-01-Youth matters in London: Mental health, addiction and homelessness. European Psychiatry. 2011 Jan 1;26:1965.	Wrong publication
30	French R, Reardon M, Smith P. Engaging with a mental health service: Perspectives of at-risk youth. Child and Adolescent Social Work Journal. 2003 Dec 1;20(6):529-48.	Low Quality
31	Freund PD, Hawkins DW. What street people reported about service access and drug treatment. Journal of Health & Social Policy. 2004 Jun 7;18(3):87-93.	Wrong study design
32	Grazioli VS, Collins SE, Daeppen JB, Larimer ME. 498 Perceptions Of Alcoholics Anonymous Among Chronically Homeless Individuals With Alcohol-use Disorders. Alcoholism: Clinical & Experimental Research. 2013 Jun 1;37:135A.	Wrong study design
33	Håkanson C, Christiansen M, Ekstedt M, Sandberg J, Kenne Sarenmalm E, Öhlén J. Experience-based knowledge in the care of homeless people with severe illness and complex care needs. In Palliative Medicine: A Multiprofessional Journal 2014 (Vol. 28, No. 6, pp. 789-789).	Wrong population
34	Haldenby AM, Berman H, Forchuk C. Homelessness and health in adolescents. Qualitative Health Research. 2007 Nov;17(9):1232-44.	Wrong intervention
35	Haley RJ, Woodward KR. Perceptions of individuals who are homeless: healthcare access and utilization in San Diego. Advanced Emergency Nursing Journal. 2007 Oct 1;29(4):346-55.	Wrong intervention

36	Harding J, Kirk R. The housing and support needs of teenage mothers. Housing, Care and Support. 2004 Sep 1;7(3):16-9.	Wrong intervention
37	Hawkins EJ, Danner AN, Malte CA, Painter JM, Lott AM, Baer JS. Feasibility of a care management approach for complex substance use disorders and high acute services utilization. Journal of substance abuse treatment. 2018 Sep 1;92:100-8.	Wrong population
38	Hemington J. Homeless clients' perceptions of differences between Continuum of Care and Housing First programs.	Wrong publication
39	Joanne Neale BA, Hons CQ, MA D, Kennedy C. Good practice towards homeless drug users: research evidence from Scotland. Health & social care in the community. 2002 May;10(3):196-205.	Low Quality
40	Jost JJ, Levitt AJ, Hannigan A, Barbosa A, Matuza S. Promoting consumer choice and empowerment through tenant choice of supportive housing case manager. American Journal of Psychiatric Rehabilitation. 2014 Jan 1;17(1):72-91.	Wrong intervention
41	Jost JJ, Levitt AJ, Hannigan A, Barbosa A, Matuza S. Promoting consumer choice and empowerment through tenant choice of supportive housing case manager. American Journal of Psychiatric Rehabilitation. 2014 Jan 1;17(1):72-91.	Wrong publication- Abstract
42	Kidd SA, Kirkpatrick H, George L. Getting to know Mark, a homeless alcohol-dependent artist, as he finds his way out of the river. Addiction Research & Theory. 2011 Apr 1;19(2):102-11.	Low Quality
43	Kim MM, Swanson JW, Swartz MS, Bradford DW, Mustillo SA, Elbogen EB. Healthcare barriers among severely mentally ill homeless adults: Evidence from the five-site health and risk study. Administration and Policy in Mental Health and Mental Health Services Research. 2007 Jul 1;34(4):363-75.	Wrong study design
44	Kirst M, Zerger S, Harris DW, Plenert E, Stergiopoulos V. The promise of recovery: narratives of hope among homeless individuals with mental illness participating in a Housing First randomised controlled trial in Toronto, Canada. BMJ open. 2014 Mar 1;4(3):e004379.	Low Quality
45	Kissman K. Respite from stress and other service needs of homeless families. Community Mental Health Journal. 1999 Jun 1;35(3):241-9.	Low Quality
46	Klitzing SW. Coping with chronic stress: Leisure and women who are homeless. Leisure Sciences. 2003 Apr 1;25(2-3):163-81.	Wrong intervention
47	Klodawsky F, Aubry T, Nemiroff R, Bonetta C, Willis A. What happens over time: Researching homelessness longitudinally. Canadian Journal of Urban Research. 2007 Jul 1;16(1):93-111.	Wrong intervention
48	Knight KR, Lopez AM, Comfort M, Shumway M, Cohen J, Riley ED. Single room occupancy (SRO) hotels as mental health risk environments among impoverished women: The intersection of policy, drug use, trauma, and urban space. International Journal of Drug Policy. 2014 May 1;25(3):556-61.	Wrong intervention
49	Koester S, Mueller SR, Raville L, Langegger S, Binswanger IA. Why are some people who have received overdose education and naloxone reticent to call Emergency Medical Services in the event of overdose?. International Journal of Drug Policy. 2017 Oct 1;48:115-24.	Irrelevant outcomes
50	Krüsi A, Fast D, Small W, Wood E, Kerr T. Social and structural barriers to housing among street-involved youth who use illicit drugs. Health & social care in the community. 2010 May;18(3):282-8.	Wrong intervention
51	Kryda AD, Compton MT. Mistrust of outreach workers and lack of confidence in available services among individuals who are chronically street homeless. Community Mental Health Journal. 2009 Apr 1;45(2):144.	Wrong intervention
52	Labrecque J, Walsh CA. Homeless women's voices on incorporating companion animals into shelter services. Anthrozoös. 2011 Mar 1;24(1):79-95.	Wrong intervention
53	Lamanna D, Stergiopoulos V, Durbin J, O'campo P, Poremski D, Tepper J. Promoting continuity of care for homeless adults with unmet health needs: The role of brief interventions. Health & social care in the community. 2018 Jan;26(1):56-64.	Wrong population

54	Lemos, Adriana Guerra Abreu, Moraes, Mayara Muniz Bastos, Alves, Daniel Gonçalves, Halpern, Elizabeth Espindola, & Leite, Ligia Costa. (2014). Evasão nas unidades de acolhimento: discutindo seus significados. Psicologia & Sociedade, 26(3), 594-602.	Wrong population
55	Lem M, Coe JB, Haley DB, Stone E. Effects of companion animal ownership among Canadian street-involved youth: A qualitative analysis. J. Soc. & Soc. Welfare. 2013;40:285.	Wrong intervention
56	Lindsey EW. Mothers' perceptions of factors influencing the restabilization of homeless families. Families in society. 1996 Apr;77(4):203-15.	Wrong intervention
57	Little M. The voices of mindfulness, attachment-related strategies and the mother-child relationship.	Wrong publication
58	Lorelle S. A phenomenological study of children's experiences while families receive services from a homeless agency. Old Dominion University; 2010.	Wrong publication
59	Lovell E, Hutchison B, Cabulagan KA, McMullin J, Child C. Homelessness and the High Performance Cycle: A New Lens for Studying Exit Strategies. Journal of Social Service Research. 2015 Aug 8;41(4):508-29.	Wrong intervention
60	Mack-Sanders SL. Collaborative action for women's shelter programs improvement: An outcome-based evaluative case study (Doctoral dissertation, Capella University).	Wrong publication
61	MacLellan J, Surey J, Abubakar I, Stagg HR, Mannell J. Using peer advocates to improve access to services among hard-to-reach populations with hepatitis C: a qualitative study of client and provider relationships. Harm reduction journal. 2017 Dec;14(1):76.	Wrong population
62	Macmaster SA. Social Service Delivery Preferences Among African American Women Who Use Crack Cocaine: What Women Say They Need Before They Can Be Open to HIV Prevention Services?. Journal of HIV/AIDS & Social Services. 2006 Dec 19;5(3-4):161-79.	Wrong population
63	Macnaughton E, McCay B. Engaging people with early psychosis in housing first. InEARLY INTERVENTION IN PSYCHIATRY 2012 Oct 1 (Vol. 6, pp. 98-98). 111 RIVER ST, HOBOKEN 07030-5774, NJ USA: WILEY-BLACKWELL.	Wrong publication
64	Macnaughton E, Nelson G, Goering P. Bringing politics and evidence together: policy entrepreneurship and the conception of the At Home/Chez Soi Housing First Initiative for addressing homelessness and mental illness in Canada. Social Science & Medicine. 2013 Apr 1;82:100-7.	Wrong publication
65	Mancini MA, Wyrick-Waugh W. Consumer and practitioner perceptions of the harm reduction approach in a community mental health setting. Community mental health journal. 2013 Feb 1;49(1):14-24.	Wrong population
66	Mantler T, Wolfe B. A rural shelter in Ontario adapting to address the changing needs of women who have experienced intimate partner violence: a qualitative case study. Rural & Remote Health. 2017 Jan 1;17.	Wrong population
67	Martin D, Sweeney J, Cooke J. Views of teenage parents on their support housing needs. Community Practitioner. 2005 Nov 1;78(11):392.	Wrong intervention
68	Marzol RM, Bonafé L, Yunes MA. As perspectivas de crianças e adolescentes em situação de acolhimento sobre os cuidadores protetores. Psico. 2012;43(3):5.	Wrong geographical setting
69	Mastropieri B, Schussel L, Forbes D, Miller L. Inner resources for survival: Integrating interpersonal psychotherapy with spiritual visualization with homeless youth. Journal of religion and health. 2015 Jun 1;54(3):903-21.	Wrong study design
70	Matarese MT, van Nijnatten C. Making a case for client insistence in social work interaction. Discourse Processes. 2015 Nov 17;52(8):670-88.	Wrong intervention
71	Mattson S, Shearer N, Long C. Exploring telehealth opportunities in domestic violence shelters. Journal of the American Academy of Nurse Practitioners. 2002 Oct;14(10):465-70.	Wrong intervention
72	Mayock P, Corr ML, O'sullivan E. Homeless young people, families and change: Family support as a facilitator to exiting homelessness. Child & Family Social Work. 2011 Nov 1;16(4):391-401.	Wrong intervention

73	Miller AB. Violence and abuse in society: Understanding a global crisis. ABC-CLIO; 2012.	Wrong publication
74	Munson MR, Lox JA. Clinical social work practice with former system youth with mental health needs: Perspective of those in need. Clinical Social Work Journal. 2012 Jun 1;40(2):255-60.	Wrong study design
75	Murphy NF. Developing evidence-based effective principles for working with homeless youth: a developmental Evaluation of the Otto Bremer Foundation's support for collaboration among agencies serving homeless youth.	Wrong publication
76	Neale J, Stevenson C. Positive and negative features of a computer assisted drug treatment program delivered by mentors to homeless drug users living in hostels. Journal of substance abuse treatment. 2014 Oct 1;47(4):258-64.	Wrong intervention
77	Oliva EM, Nevedal A, Lewis ET, McCaa MD, Cochran MF, Konicki PE, Davis CS, Wilder C. Patient perspectives on an opioid overdose education and naloxone distribution program in the US Department of Veterans Affairs. Substance abuse. 2016 Jan 2;37(1):118-26.	Low Quality
78	Oliver V, Cheff R. Sexual health: the role of sexual health services among homeless young women living in Toronto, Canada. Health promotion practice. 2012 May;13(3):370-7.	Wrong intervention
79	Osilla KC, Kennedy DP, Hunter SB, Maksabedian E. Feasibility of a computer-assisted social network motivational interviewing intervention for substance use and HIV risk behaviors for housing first residents. Addiction science & clinical practice. 2016 Dec;11(1):14.	Wrong intervention
80	Padgett DK, Henwood B, Abrams C, Davis A. Engagement and retention in services among formerly homeless adults with co-occurring mental illness and substance abuse: Voices from the margins. Psychiatric rehabilitation journal. 2008;31(3):226.	Wrong intervention
81	Parsell C, Petersen M, Moutou O. Single-site supportive housing: tenant perspectives. Housing Studies. 2015 Nov 17;30(8):1189-209.	Wrong study design
82	Paudyal V, Stewart D, MacLure K, Buchanan C, Wilson L, MacLeod J. Expectations, beliefs, behaviours and sources of information on prescribed medicines by homeless patients. InINTERNATIONAL JOURNAL OF CLINICAL PHARMACY 2016 Apr 1 (Vol. 38, No. 2, pp. 480-480). VAN GODEWIJCKSTRAAT 30, 3311 GZ DORDRECHT, NETHERLANDS: SPRINGER.	Wrong publication
83	Paudyal V, Stewart D, Maclure K, Buchanan C, Macleod J, Wilson L. perspectives of homeless patients on their prescribed medicines: 0004. International Journal of Pharmacy Practice. 2015 Oct 1;23:6-7.	Low Quality
84	Peixoto AS, Machado C. " A very good family…": Voices of women who have lived in a shelter for domestic abuse victims. Psicologia Educação Cultura. 2010;14(2):373-99.	Wrong study design
85	Piat M, Ricard N, Sabetti J, Beauvais L. Building life around foster home versus moving on: The competing needs of people living in foster homes. Psychiatric rehabilitation journal. 2008;32(1):32.	Wrong population
86	Polvere L, Macnaughton E, Piat M. Participant perspectives on housing first and recovery: Early findings from the At Home/Chez Soi project. Psychiatric Rehabilitation Journal. 2013 Jun;36(2):110.	Low Quality
87	Pritchard AJ, Jordan CE, Jones L. A qualitative comparison of battered women's perceptions of service needs and barriers across correctional and shelter contexts. Criminal Justice and Behavior. 2014 Jul;41(7):844-61.	Wrong population
88	Quimby E. Homeless clients' perspectives on recovery in the Washington, DC, dual diagnosis project. Contemporary Drug Problems. 1995 Jun;22(2):265-89.	Low Quality
89	Quine S, Kendig H, Russell C, Touchard D. Health promotion for socially disadvantaged groups: the case of homeless older men in Australia. Health Promotion International. 2004 Jun 1;19(2):157-65.	Wrong intervention
90	Robbins PC, Callahan L, Monahan J. Perceived coercion to treatment and housing satisfaction in housing-first and supportive housing programs. Psychiatric Services. 2009 Sep;60(9):1251-3.	Wrong study design
91	Rosenfeld SL, Keenan PM, Fox DJ, Chase LH, Melchiono MW, Woods ER. Youth perceptions of	Low Quality

	comprehensive adolescent health services through the Boston HAPPENS program. Journal of Pediatric Health Care. 2000 Mar 1;14(2):60-7.	
92	Roy É, Denis V, Gutiérrez N, Haley N, Morissette C, Boudreau JF. Evaluation of a media campaign aimed at preventing initiation into drug injection among street youth. Drugs: education, prevention and policy. 2007 Jan 1;14(5):401-14.	Wrong study design
93	Russell L. What happens next? A qualitative program evaluation of paradise adolescent homes and adolescent girls' transition into independence (Doctoral dissertation, The Wright Institute).	Wrong publication
94	Stahler GJ, Cohen E, Greene MA, Shipley TE, Bartelt D. A qualitative study of treatment success among homeless crack-addicted men: definitions and attributions. Contemporary Drug Problems. 1995 Jun;22(2):237-64.	Low Quality
95	Stergiopoulos V, O'Campo P, Gozdzik A, Jeyaratnam J, Corneau S, Sarang A, Hwang SW. Moving from rhetoric to reality: adapting Housing First for homeless individuals with mental illness from ethno-racial groups. BMC Health Services Research. 2012 Dec;12(1):345.	Low Quality
96	Stewart M, Reutter L, Letourneau N. Support intervention for homeless youths. Canadian Journal of Nursing Research Archive. 2007 Sep 15;39(3).	Wrong publication
97	Tsai J, Bond GR, Salyers MP, Godfrey JL, Davis KE. Housing preferences and choices among adults with mental illness and substance use disorders: A qualitative study. Community Mental Health Journal. 2010 Aug 1;46(4):381-8.	Wrong intervention
98	Ungar M, Ikeda J. Rules or no rules? Three strategies for engagement with young people in mandated services. Child and Adolescent Social Work Journal. 2017 Jun 1;34(3):259-67.	Wrong population
99	Vance D. Barriers to use of services by older homeless people. Psychological reports. 1994 Dec;75(3):1377-8.	Wrong publication
100	Waldbrook N. Formerly homeless, older women's experiences with health, housing, and aging. Journal of women & Aging. 2013 Oct 1;25(4):337-57.	Wrong intervention
101	Waldbrook N. Exploring opportunities for healthy aging among older persons with a history of homelessness in Toronto, Canada. Social Science & Medicine. 2015 Mar 1;128:126-33.	Irrelevant outcomes
102	Wallace B, Barber K, Pauly BB. Sheltering risks: Implementation of harm reduction in homeless shelters during an overdose emergency. International Journal of Drug Policy. 2018 Mar 1;53:83-9.	Wrong population
103	Walsh CA, Hanley J, Ives N, Hordyk SR. Exploring the experiences of newcomer women with insecure housing in Montréal Canada. Journal of International Migration and Integration. 2016 Aug 1;17(3):887-904.	Wrong intervention
104	Walsh CA, Rutherford GE, Kuzmak N. Characteristics of home: Perspectives of women who are homeless. The Qualitative Report. 2009;14(2):299-317.	Wrong intervention
105	Weinstein LC, LaNoue M, Hurley K, Sifri R, Myers R. Using concept mapping to explore barriers and facilitators to breast cancer screening in formerly homeless women with serious mental illness. Journal of health care for the poor and underserved. 2015;26(3):908-25.	Wrong intervention
106	Williams FA. Short-Term Emergency Shelter Care for Chronically Homeless Urban African American Adolescents (Doctoral dissertation, Walden University).	Wrong publication
107	Voronka J, Wise Harris D, Grant J, Komaroff J, Boyle D, Kennedy A. Un/helpful help and its discontents: Peer researchers paying attention to street life narratives to inform social work policy and practice. Social Work in Mental Health. 2014 May 4;12(3):249-79.	Low Quality
108	Yanos PT, Barrow SM, Tsemberis S. Community integration in the early phase of housing among homeless persons diagnosed with severe mental illness: Successes and challenges. Community Mental Health Journal. 2004 Apr 1;40(2):133-50.	Wrong study design
